# SARS-CoV-2 Papain-Like Protease Potential Inhibitors—In Silico Quantitative Assessment

**DOI:** 10.3390/ijms22083957

**Published:** 2021-04-12

**Authors:** Adam Stasiulewicz, Alicja W. Maksymiuk, Mai Lan Nguyen, Barbara Bełza, Joanna I. Sulkowska

**Affiliations:** 1 Centre of New Technologies, University of Warsaw, Banacha 2c, 02-097 Warsaw, Poland; a.stasiulewicz@cent.uw.edu.pl (A.S.); a.maksymiuk@cent.uw.edu.pl (A.W.M.); m.nguyen@cent.uw.edu.pl (M.L.N.); b.belza@cent.uw.edu.pl (B.B.); 2 Faculty of Pharmacy, Medical University of Warsaw, Banacha 1, 02-097 Warsaw, Poland; 3 School of the Biological Sciences, University of Cambridge, 17 Mill Lane, Cambridge CB2 1RX, UK; 4 Faculty of Mathematics, Informatics and Mechanics, University of Warsaw, Banacha 2, 02-097 Warsaw, Poland; 5 College of Inter-Faculty Individual Studies in Mathematics and Natural Sciences, University of Warsaw, Banacha 2c, 02-097 Warsaw, Poland

**Keywords:** SARS-CoV-2, COVID-19, coronavirus, PLpro, papain-like protease, UCH-L1, virtual screening, docking, pharmacophore

## Abstract

Severe acute respiratory syndrome coronavirus 2 (SARS-CoV-2) encodes the papain-like protease (PLpro). The protein not only plays an essential role in viral replication but also cleaves ubiquitin and ubiquitin-like interferon-stimulated gene 15 protein (ISG15) from host proteins, making it an important target for developing new antiviral drugs. In this study, we searched for novel, noncovalent potential PLpro inhibitors by employing a multistep in silico screening of a 15 million compound library. The selectivity of the best-scored compounds was evaluated by checking their binding affinity to the human ubiquitin carboxy-terminal hydrolase L1 (UCH-L1), which, as a deubiquitylating enzyme, exhibits structural and functional similarities to the PLpro. As a result, we identified 387 potential, selective PLpro inhibitors, from which we retrieved the 20 best compounds according to their IC_50_ values toward PLpro estimated by a multiple linear regression model. The selected candidates display potential activity against the protein with IC_50_ values in the nanomolar range from approximately 159 to 505 nM and mostly adopt a similar binding mode to the known, noncovalent SARS-CoV-2 PLpro inhibitors. We further propose the six most promising compounds for future in vitro evaluation. The results for the top potential PLpro inhibitors are deposited in the database prepared to facilitate research on anti-SARS-CoV-2 drugs.

## 1. Introduction

Due to the alarming spread levels and rising infection numbers, on 11 March 2020, the World Health Organization (WHO) declared coronavirus disease 2019 (COVID-19) as a world health emergency and characterized it as a pandemic [1]. Severe acute respiratory syndrome coronavirus 2 (SARS-CoV-2) was identified as pathogen causing COVID-19 [2]. People infected with SARS-CoV-2 can suffer from mild symptoms such as high fever, cough, and fatigue, but the virus can also cause acute respiratory difficulties, multiple organ failure, and death. The elderly and people with comorbidities are especially at risk of a severe course of the disease [3]. As of March 2021, more than 2.5M people have died from COVID-19 and more than 114M confirmed infection cases have been reported around the world [4,5]. As the disease has a substantial impact on global health and significantly affects social and economic aspects, the scientific community has put great effort toward developing new treatments.

SARS-CoV-2 is an enveloped positive-strand RNA virus from the Coronaviridae family [6]. In the infection cycle, viral polypeptides (pp1a and pp1ab) are translated. They have to be cleaved by a protease to become functional peptides [7,8]. In SARS-CoV-2, two proteases were identified as essential in the cleavage process—namely, main protease (Mpro) and papain-like protease (PLpro). As they determine successful viral replication, Mpro and PLpro became potential drug targets [9,10,11]. Inhibition of viral proteases crucial for polypeptide processing has been reported as a successful strategy for the treatment of other viruses—hepatitis C virus (HCV) and human immunodeficiency virus (HIV) [12,13].

Beyond viral polypeptides cleavage function, PLpro also has the ability to reverse post-translational modifications of host proteins. It can hydrolyze a bond between a host protein and ubiquitin (Ub) or protein of interferon-stimulated gene 15 (ISG15) [14]. Coronavirus PLpro recognizes the LXGG sequence as the cleavage target. The same sequence is recognized by deubiquitinating enzymes [8,15]. Structural similarities to other viral ubiquitin-specific proteases have been reported [16]. A ubiquitin-like protein ISG15, the product of interferon-stimulated gene, consists of a C-terminal LRLRGG sequence, which makes it a PLpro cleavage target. ISGylation and ubiquitination events are prominent elements of the innate immune response, which have to be broken by the virus for successful infection [7,16]. Both ISG15 and Ub proteins are substantial mediators and regulators in antiviral defense [14]. By modifying host proteins, PLpro suppresses the immune system response [7]. The above functions, along with polyprotein processing, make PLpro an ideal molecular target for potential anti-SARS-CoV-2 drugs.

Structural similarities to PLpro can be found in a human protein—ubiquitin carboxy-terminal hydrolase L1 (UCH-L1). UCH-L1 recognizes the same target sequence and exhibits functional similarity to PLpro. It belongs to deubiquitinating enzymes (DUBs, deubiquitylases) family and plays a significant role in neural protein homeostasis by removing ubiquitin or adding it to proteasome-degradation-destined proteins [17]. Mutations in UCH-L1 cause neural disorders, including Parkinson’s disease [18]. As deregulation of UCH-L1 level can lead to Alzheimer’s disease [19] and some types of cancer [20], UCH-L1 is frequently used in research as a drug target [21]. UCH-L1, as well as other C-terminal hydrolases (UCHs family members), possesses a nontrivial topology; its protein backbone forms a knot (when pulled by termini tied) [22,23]. Due to UCH-L1’s important function, it is clear that the effect of potential drug binding to this protein has to be taken into account when new inhibitors for SARS-CoV-2 PLpro are designed. Both human and viral deubiquitinating enzymes have to be evaluated to identify safe anti-SARS-CoV-2 drugs.

Because SARS-CoV-2 PLpro is necessary for viral replication, as well as for suppression of the host immune response, it is a promising molecular target. As such, it has already been put in a spotlight by the scientific community. This includes research on its structure [24,25,26], functions [11,27], and similarity to PLpro from other related coronaviruses, most notably SARS-CoV [28]. The search for PLpro inhibitors has already begun. So far, most studies focus on trying to utilize previously developed noncovalent SARS-CoV inhibitors or their derivatives [7,24,29] or to design specific, covalent inhibitors [30,31]. However, covalent protease inhibitors come with risks of toxicity due to high reactivity and, in some cases, low selectivity and nonspecific binding off the target [32,33,34]. In this context, it may be favorable to design noncovalent PLpro inhibitors. There is a considerable amount of data on such compounds with regards to SARS-CoV PLpro, which will facilitate the design of analogical inhibitors for the novel coronavirus, as these two strains share considerable similarity in terms of PLpro structure [24]. Alas, the recently developed and studied SARS-CoV-2 PLpro inhibitors exhibit only moderate binding affinity toward this enzyme [7,24,35,36]. Furthermore, they lack consideration of potential toxicity, most importantly in terms of potential off binding to similar deubiquitinating enzymes, most notably to UCH-L1. Thus, a new, more comprehensive approach should be taken to design novel PLpro inhibitors with potentially higher binding affinities and a low toxicity.

In this study, we were searching for novel, potent, noncovalent, and selective PLpro inhibitors. Such compounds would have higher affinity to SARS-CoV-2 PLpro than up-to-date known inhibitors as well as low affinity toward human UCH-L1 to reduce the risk of toxicity. In order to achieve these goals, we conducted comprehensive in silico screening, which is an effective and relatively fast route in modern drug design. We put an emphasis on the high quality of our predictions. Thus, we extensively validated used techniques, to ascertain that they are suitable for this specific project. As a result, we employed a broad range of computational methods, merging both ligand-based and structure-based ones. Initially, we screened a large library of over 15M drug-like compounds using mixed pharmacophores. Then, we evaluated binding affinities of selected molecules toward SARS-CoV-2 PLpro with molecular docking and binding energy calculations. The potential PLpro inhibitors were then tested for their ability to bind to human UCH-L1. Finally, we conducted an in-depth analysis of the selected best compounds’ chemical structures and binding modes and evaluated their toxicity. We identified nearly 1000 compounds, including six with very high probability to be potential selective PLpro inhibitors. These results are deposited in a publicly available database to allow future investigation to overcome the COVID-19 pandemics.

## 2. Results and Discussion

### 2.1. Overview of SARS-CoV-2 PLpro Structure and Its Known Inhibitors

**Figure 1:**
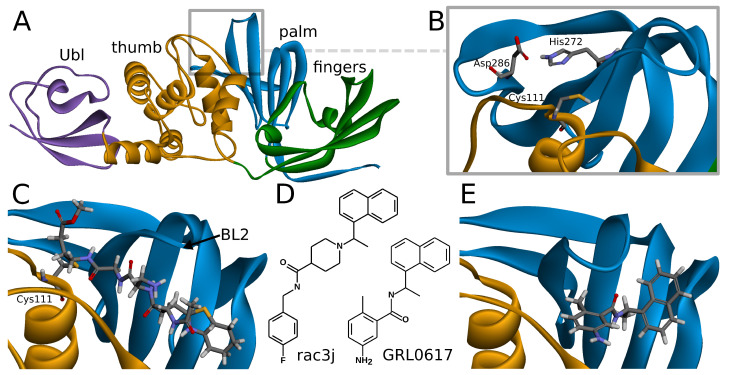
Overview of the SARS-CoV-2 PLpro structure and inhibitors. (**A**) Tertiary structure of PLpro and its division into two main parts: the ubiquitin-like (Ubl) domain (purple), and the catalytic domain, which may be further divided into three subdomains: thumb (orange), palm (blue), and fingers (green). (**B**) PLpro active site at the interface between palm and thumb. Three amino acids of the catalytic triad—Cys111, His272, and Asp286 are depicted in stick representation (PDB ID: 7jn2). (**C**) PLpro with covalent peptide inhibitor VIR250 bound to Cys111, shown in stick representation. Part of the inhibitor exceeds beyond the active site and is placed under the blocking loop 2 (BL2) (PDB ID: 6wuu). (**D**) Structural formulas of the representatives of the two main classes of SARS-CoV-2 noncovalent inhibitors—GRL0617 and rac3j. (**E**) PLpro with noncovalent inhibitor GRL0617. Such compounds bind under the BL2, just outside the active site (PDB ID: 7jir).

SARS-CoV-2 PLpro is a monomeric enzyme that may be divided into two main domains—the catalytic domain and the ubiquitin-like (Ubl) domain. The first one is the interesting part in terms of enzymatic functions, as well as inhibiting the protein. It may be further divided into three subdomains: thumb, palm, and fingers (Figure 1A). The active site is located between palm and thumb, utilizing three main residues, called the catalytic triad: Cys111, His272, and Asp286 (Figure 1B) [24].

Although SARS-CoV-2 PLpro is a relatively short known protein, its great significance for the virus, as well as being an immensely valid molecular target for novel potential drugs, led to great attention on it in the scientific world. Thus, multiple crystal structures of this enzyme are already available, including apo-protein [24,25] as well as structures of PLpro with ubiquitin, ISG15 [35] or inhibitors [24,25,30]. There are two main classes of SARS-CoV-2 PLpro inhibitors, with both solved tertiary structures of protein–inhibitor complexes and results of in vitro studies regarding their binding affinities.

Covalent inhibitors represent one of the most important types of compounds studied so far. In this group, the main direction seems to be focused on the peptide scaffolds [30]. There are also other proposals for nonpeptide, covalent inhibitors, such as ebselen [37], its derivatives [31], and disulfiram [38]. In the case of the peptide inhibitors, the covalent bond is formed with one of the amino acids of the catalytic triad—Cys111. As the structures of those compounds form quite long chains, the large part of the molecule is placed out of the active site and lies under the blocking loop 2 (BL2) in the palm subdomain (Figure 1C) [30].

Noncovalent PLpro inhibitors are the second main type of potential anti-SARS-CoV-2 drugs. In this group, the most significant and the most extensively studied compounds include inhibitors known to act on SARS-CoV PLpro and their derivatives. Such a direction is reasonable due to the high sequential similarity (90%) and identity (83%) of PLpro between SARS-CoV and SARS-CoV-2 [24]. The SARS-CoV PLpro noncovalent inhibitors are in a big part members of one structural group of compounds, namely, the derivatives of N-[1-(naphthalen-1-yl)ethyl]benzamide (e.g., GRL0617) [7]. Other proposals include, most notably, derivatives of N-benzyl-1-[1-(naphthalen-1-yl)ethyl]piperidine-4-carboxamide (e.g., rac3j), with a different arrangement of the scaffold in the center of the molecule [35] (Figure 1D). A recently developed class of GRL0617 derivatives retains its central N-ethylbenzamide part, but replaces the naphthyl group with a 2-phenylthiophene moiety [36]. Importantly, the noncovalent inhibitors do not bind at the active site, but instead nearby, below the BL2, similarly to the part of the structure of the peptide, covalent inhibitors (Figure 1E). This binding site is placed at the interface between palm and thumb. Residues from both of these subdomains take part in forming protein–ligand interactions. While most of the site is quite rigid, the crucial BL2 is a flexible loop, exhibiting considerable induced fit effects. Our analysis of available structures as well as conformations from explicit solvent molecular dynamics simulations implies that the conformation of the BL2 varies significantly, depending on the presence and the type of the inhibitor. Thus, not every tertiary structure of PLpro is equally suitable for computer-aided drug design and there is a need to rationally select applicable ones.

At present, new SARS-CoV-2 PLpro crystal structures are constantly determined, and to date, there are over 30 available at the Protein Data Bank (PDB) (Supplementary Table S1). From the structure-based drug design perspective, the most crucial factor is the presence and the type of ligand at the binding site of interest. Thus, the PLpro structures may be divided into a few main groups. The first one includes crystals with the apo-enzyme, namely PDB IDs: 6wrh, 6wzu, 6xg3 [24], 7cjd [25], 6w9c, 7d47, 7d6h, and 7nfv. Because of the induced fit effect at the BL2, the apo conformations are in most cases the least useful ones for an application in structure-based computational methods. The second type of PLpro structures contains natural ligands, namely ubiquitin (PDB ID: 6xaa [35]) and ISG15 (PDB IDs: 6yva [11] and 6xa9 [35]). Those structures should intuitively be more suitable than the apo ones. However, a more detailed analysis of the BL2 conformation and our validation show that the structures with ubiquitin or ISG15 are also not sufficient for the in silico screening. The third group contains PLpro with covalent, peptide inhibitors (PDB IDs: 6wuu and 6wx4 [30]). Covalent inhibitors, apart from an obvious covalent bond, present a distinct binding mode compared to the noncovalent compounds. Once again, this is especially visible in the BL2 conformation, therefore limiting these crystal structures utility for noncovalent inhibitors’ design. The fourth group includes PLpro with cocrystallized GRL0617 or its close derivatives, namely PDB IDs: 7jir, 7jit, 7jiv, 7jiw [24], 7cmd [25], 7jn2, 7koj, 7kok, 7kol, 7krx, 7jrn, and 7cjm. These structures are well suited for the in silico screening. All the ligands in this group retain the N-[1-(naphthalen-1-yl)ethyl]benzamide scaffold of the GRL0617, varying only in the substituents attached to the phenyl group. Thus, the conformations of the BL2 remain almost exactly the same, and the aforementioned PDB entries exhibit only slight differences. Selection of one representative PLpro structure from this set is therefore a sufficient strategy. The next group contains GRL0617 derivatives with N-ethylbenzamide scaffold retained but with the naphthyl group replaced with a 2-phenylthiophene moiety (PDB IDs: 7lbr, 7lbs, 7llf, 7llz, and 7los [36]). However, the altered fragment of the inhibitors is placed between the BL2 and the rest of the palm subdomain and in turn does not cause any significant BL2 conformational rearrangements compared to GRL0617-bound structures. The next type of the PLpro structure is the PDB ID: 7e35 with the derivative of rac3j. As the inhibitor cocrystallized in this entry possesses a distinct scaffold than GRL0617, the conformation of BL2 varies slightly in this structure. Thus, it may be utilized alternatively to or together with the aforementioned noncovalent inhibitor-bound PLpro structures in order to design potentially more diverse compounds. The last PLpro structure type is the PDB ID: 7m1y with ebselen. However, this compound is bound at a distinct site, and thus this PLpro conformation is not suitable for design of noncovalent inhibitors, similarly to apo-structures.

### 2.2. Overview of Human UCH-L1 and Its Similarity to SARS-CoV-2 PLpro

**Figure 2:**
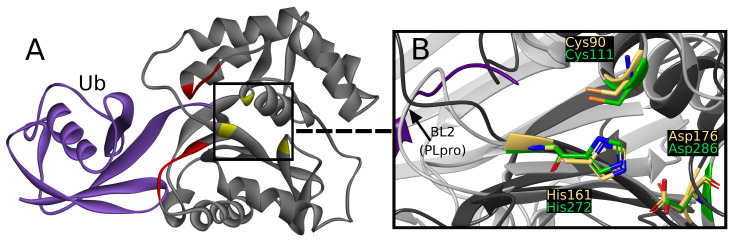
(**A**) Overview of UCH-L1 structure (PDB ID: 3kw5) with ubiquitin (purple), ubiquitin binding sites (red), and catalytic triad (yellow). (**B**) UCH-L1 active site (dark grey ribbon and yellow sticks) superimposed on PLpro active site (light grey ribbon and green sticks) from PDB structures 3kw5 and 7jn2, respectively.

UCH-L1 is a proteolytic enzyme that hydrolyzes the peptide bond with glycine at ubiquitin’s C-terminus. Thus far, eight crystallographic structures have been solved, one of which was cocrystallized with a covalent, peptide inhibitor [39] and other three with ubiquitin, which is considered to interact with two sites created with residues 5–10 and 211–216 (Figure 2A) [40].

In native state, UCH-L1 exists as a monomer. However, it was shown that this protein, unlike other UCH family members, can create an asymmetric homodimer while exhibiting additional ubiquitin ligase activity [41,42]. Its secondary structure comprises, similarly to UCH-L3, a helix-β-helix sandwich fold consisting of a right lobe—five α-helices, and left lobe—two α-helices and six β-strands [41]. The highly folded protein backbone creates the 5_2_ knot with a core length of 215 amino acids (residues 5-219), and a slipknot 3_1_ containing 159 amino acids (residues 6-164) [43,44].

The active site is created by Cys90, His161, and Asp176, together called the catalytic triad [45]. The catalytic triad of UCHs shows close resemblance to PLpro active site (Figure 2B) [46]. To the best of our knowledge, a comparative study of UCH-L1 and SARS-CoV-2 PLpro has not yet been conducted. Middle East respiratory syndrome coronavirus (MERS-CoV) PLpro and human UCH-L3 have been previously used together in experimental work in inhibitor research [47].

While superimposing PLpro and UCH-L1 structures by catalytic triad, the resemblance of β-strands beneath PLpro BL2, α-helices that include Cys90, and loops above active site (Figure 2B) can be seen. The similarity is sufficient to consider the risk of nonselectivity, although existing differences create possibilities of designing selective PLpro inhibitors.

### 2.3. Virtual Screening Workflow

**Figure 3:**
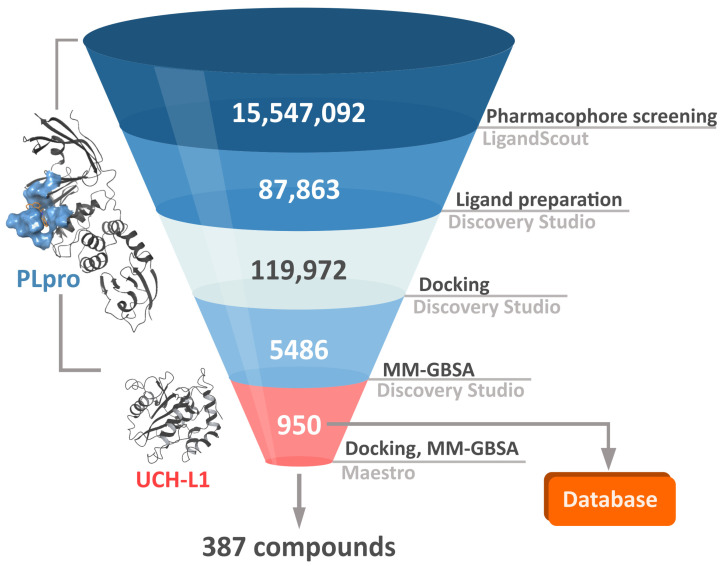
The computational workflow employed in the screening campaign. The procedure consisted of several steps aimed to find potential, potent SARS-CoV-2 inhibitors, using increasingly accurate in silico methods. First, we conducted pharmacophore screening of over 15M compounds in LigandScout. Then, we proceeded with docking and molecular mechanics–generalized Born and surface area solvation (MM–GBSA) binding energy calculations in Discovery Studio. The number of compounds obtained from the first phase initially increased from around 88 to 120 thousand due to the ligand preparation step, which included creation of possible multiple ionization states. The best 950 potential PLpro inhibitors are gathered in a database. In the last phase, we evaluated these compounds’ affinity toward human UCH-L1 in Maestro software and obtained 387 potential, selective PLpro inhibitors.

In order to both efficiently and accurately select potential PLpro inhibitors, we established a multistep workflow, including a diverse range of computational techniques. We started with relatively fast methods and, moving to the next steps, employed more accurate and time-costly ones (Figure 3). As a chemical space to screen, we picked a library of over 15 million drug-like, diverse compounds from the ENAMINE REAL database.

The first step of our workflow was to efficiently screen the above-mentioned large ligand library. For this purpose, we selected pharmacophore screening, using LigandScout [48]. As traditional ligand-based methods may have difficulties in finding novel structure groups, we utilized a mixed pharmacophore based on protein–ligand complexes obtained from the PDB. It contains descriptors derived both from a ligand’s chemical structure and from ligand–protein interactions. To further enhance our model’s ability to properly filter a broader spectrum of potential PLpro inhibitors, we merged a few such pharmacophores into a complex one. As components we used structures with SARS-CoV-2 PLpro cocrystallized with noncovalent inhibitors similar to GRL0617 [24] or covalent inhibitor [30], as well as SARS-CoV PLpro with a noncovalent inhibitor of another type than may be encountered in SARS-CoV-2 PLpro crystals present at the time, the derivative of N-benzyl-1-[1-(naphthalen-1-yl)ethyl]piperidine-4-carboxamide [49]. In this case, elements of ligand-based drug design have an advantage of being biased toward compounds similar to those exhibiting moderate successes in both SARS-CoV and SARS-CoV-2 in vitro studies. However, as we aim to find potentially superior drug candidates, the procedure able to seek also slightly different structural groups is preferable. The decision to utilize a merged model consisting of various protein–ligand complexes allowed us to obtain a less restrictive pharmacophore, capable of spotting compounds with more diverse chemical structures compared to those from crystals.

In the second phase of the screening campaign, we picked around 88,000 compounds with the best scoring function values from the previous step. In this part, we estimated binding affinities of selected compounds toward the PLpro binding site. We selected molecular docking as a semiaccurate and effective method to achieve this task. Additionally, this is a structure-based technique which allowed us to put more emphasis on the molecular target and consequentially allowed us to potentially find compounds more distinct from the PLpro inhibitors known so far. We utilized the Discovery Studio CDOCKER protocol—an accurate, rigid-protein docking program [50]. We used PDB ID: 7jn2 structure as a model of the PLpro, as it performed best during our validation. Docked compounds were scored with Jain function. Then, as a more accurate measure of binding affinity prediction, we calculated binding energies of the 5486 best-scored compounds using the molecular mechanics–generalized Born and surface area solvation (MM–GBSA) method. After this step, we left 950 potential PLpro inhibitors. As our validation showed, in this case, both Jain and MM–GBSA binding energy correlated well with experimental pIC_50_ values of PLpro ligands known so far. Thus, in order to more accurately estimate the potential inhibitors’ binding affinities, we established our own consensus function based on the multiple linear regression (MLR) for Jain and the binding energy from MM–GBSA. The combined model outperformed both its components alone when it comes to the correlation with the in vitro data. Using this model, we calculated predicted pIC_50_ values for the potential PLpro inhibitors.

The third step of the project was aimed to predict the chosen compounds’ selectivity and to filter those that potentially have a weak to no affinity toward human UCH-L1. Here, once again, we employed molecular docking. Based on the comparison of different methods and on the validation results, we decided to use Schrödinger Glide [51]. We docked 950 potential PLpro inhibitors to the model of UCH-L1 based on the PDB ID: 4jkj. For the obtained protein–ligand complexes, we calculated their binding energies with the MM–GBSA method. The results for the top candidates for PLpro inhibitors, regarding both PLpro and UCH-L1 calculations, are deposited in the database (https://plpro-inhibitors.cent.uw.edu.pl) to facilitate future work.

Finally, it is important to stress that one of the most significant difficulties in the drug design process is the potential toxicity of the drug candidates. It may be time- and money-saving to assess the potential drug’s toxicity at the early stages of the design. For this purpose, computational techniques are becoming increasingly useful [52]. In the case of the potential PLpro inhibitors, we focused mainly on the human analogous protein UCH-L1. However, there are many more factors needed to be taken into account. Thus, from the compounds with potentially low affinities toward UCH-L1, we selected 20 with lowest predicted IC_50_ values toward PLpro, based on our MLR model. Then, we assessed the potential toxicity of these selected molecules. For this purpose, we utilized the Toxicity Estimation Software Tool (TEST). We estimated the mutagenicity, developmental toxicity, and rat LD_50_ of the selected 20 compounds.

### 2.4. Validation Results

The computational methods we employed in this screening procedure may be powerful tools. However, it is important to consider their limitations. Inaccurate or semiaccurate in silico techniques may perform well when it comes to one problem but at the same time be inadequate to create a realistic model for another. This is an extremely sensitive matter when it comes to working with different proteins. Therefore, such methods require an extensive validation each time they are used for a new molecular target. We describe such validation in detail in the Methods section. However, as this is a crucial matter for such a type of an in silico project, in the next few paragraphs, we highlight the results of the validation of methods we selected for each part of our procedure.

#### 2.4.1. Pharmacophore Screening

For initial drug screening, we used a pharmacophore based simultaneously on ligand–protein interactions and the structure of inhibitors, which was chosen out of a set of many pharmacophores. To create them, we combined pharmacophores obtained from different ligand–protein complexes, because structurally diverse compounds make use of slightly varied interactions. In this way, we wanted to ensure that the pharmacophore would be able to detect compounds with different chemical structures. In order to validate the pharmacophores’ ability to spot potent inhibitors, we created a database of active compounds with low IC_50_ values and tested if a given pharmacophore can pick them up against a set of decoys.

**Figure 4:**
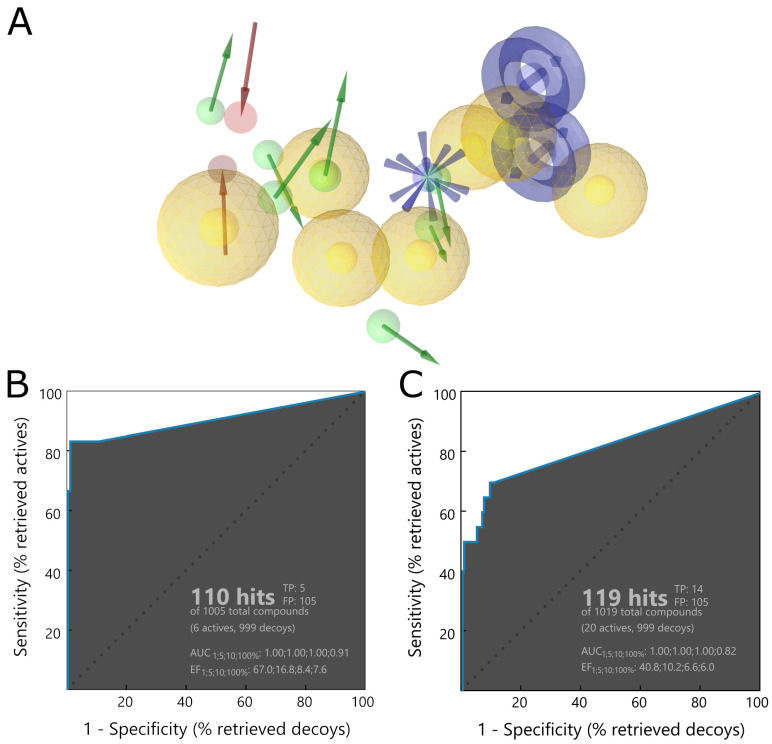
(**A**) The pharmacophore selected for screening, derived from Protein Data Bank (PDB) IDs: 7jiw, 7jn2, 4ovz, and 6wuu. Types of descriptors: green arrows—H bond donor, red arrows—H bond acceptor, yellow spheres—hydrophobic, blue rings—aromatic rings, blue star—positive ionizable. Exclusion volumes are not shown to increase the readability. Aromatic rings on the right are highly conserved between potent PLpro inhibitors because of their strong interactions with the amino acids in the binding pocket. The hydrogen bonds on the left are less important interactions, hence their positions are more varied. (**B**) Receiver operating characteristic curve obtained from the screening of the initial set of active compounds using the pharmacophore with the maximum number of omitted features set to 14. Obtained EF_1%_ = 67.0 indicates the chosen pharmacophore’s ability to distinguish potent PLpro inhibitors. (**C**) Receiver operating characteristic curve obtained from the test screening of the additional set of active compounds. The value of EF_1%_ = 40.8 confirms that the selected pharmacophore is suitable for drug screening.

Based on the validation results, we chose a pharmacophore derived from PDB IDs: 7jiw, 7jn2, 4ovz, and 6wuu with 19 descriptors presented in the Figure 4A and with 27 exclusion volumes. During validation, the pharmacophore reached the enrichment factor 1% (EF_1%_) value of 67.0, which is an excellent result. It was able to detect expected ligands as active (Figure 4B) and four of them occupied the top four places even though they were structurally different. These results prove the ability of the selected pharmacophore to spot compounds with diverse chemical structures without a bias toward only one inhibitor type.

To confirm the chosen pharmacophore’s ability to identify potent PLpro inhibitors, we conducted an additional validation step. We performed an analogical test screening, but with a bigger, more diverse set of known, active compounds with IC_50_ values below 1 μM. The pharmacophore reached a high value of EF_1%_ = 40.8, therefore proving its viability (Figure 4C).

#### 2.4.2. PLpro Binding Affinity Estimation

In our study, we used BIOVIA Discovery Studio to predict the binding affinities of the compounds selected in the previous step. This software was chosen over other tested programs (Schrödinger Maestro and Autodock Vina) because it achieved the best validation results. We employed a technique of molecular docking and then assessed the obtained poses by scoring functions and binding energy calculations. However, prior to this, we validated the software’s ability to predict correct ligand poses by redocking and cross-docking techniques. We compared ligand root-mean-square deviation (RMSD) obtained after the proteins with docking poses were superimposed on the original structures (Supplementary Table S2). With most of the redocking RMSD values under 2 Å, we can conclude that the obtained poses are valid for our further research. We observed a significant induced fit effect, so we expected that noncovalent inhibitors would reach high RMSD values after cross-docking to PLpro structures from complexes with covalent inhibitors (6wuu and 6wx4).

**Figure 5:**
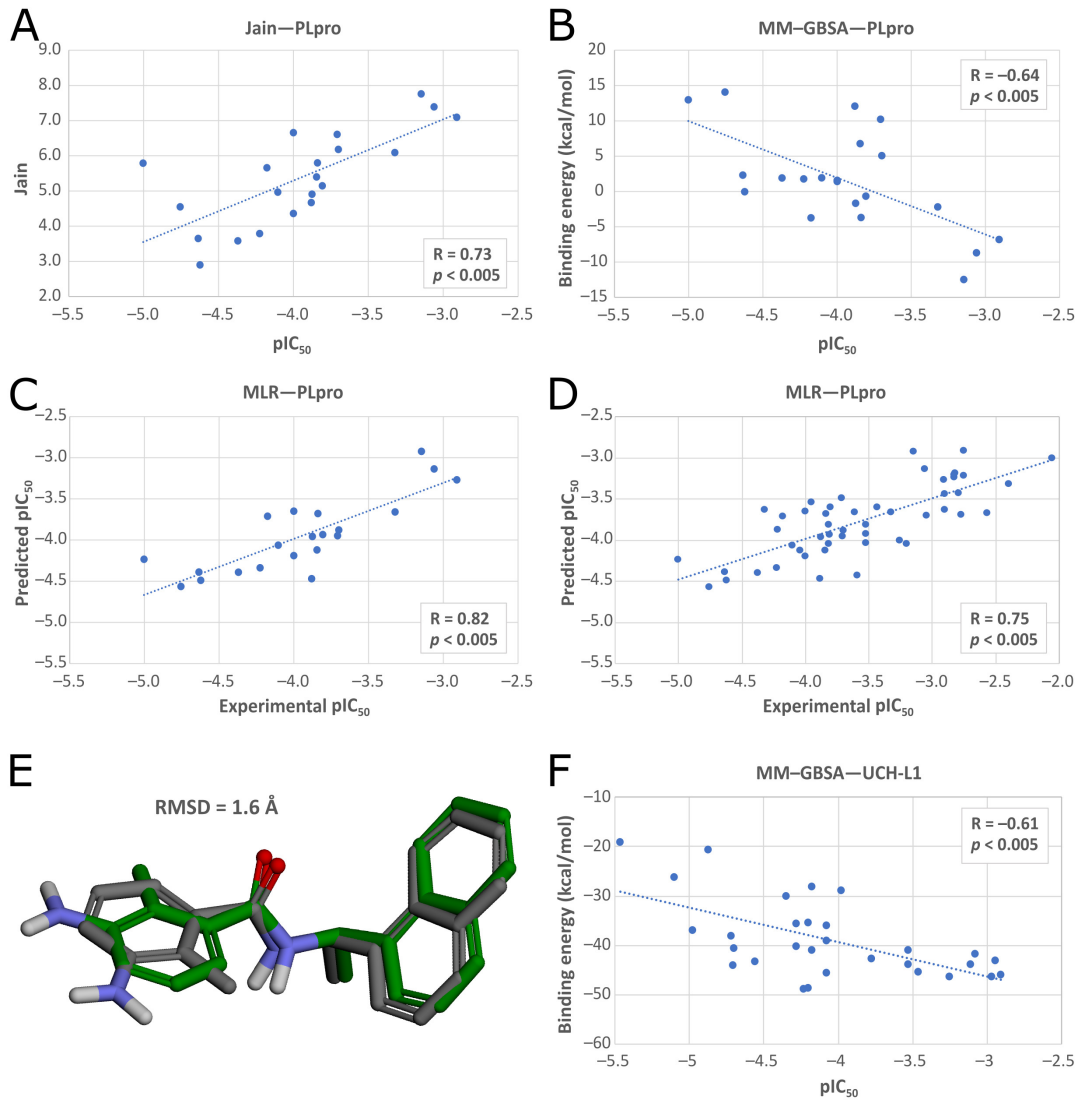
(**A**–**C**) Correlation between values of scoring functions and binding energies, and pIC_50_ values of the inhibitors docked to PLpro (PDB ID: 7jn2). (**A**) Jain scoring function. (**B**) MM–GBSA binding energy. (**C**) Multiple linear regression (MLR) model. (**D**) Analogical correlation for MLR model for the extended set of test compounds. (**E**) A comparison of poses between the PLpro inhibitor from the crystal structure (PDB ID: 7jn2, grey) and the same inhibitor after redocking (green). The naphthalene and the amide group are aligned more closely with the original ligand because of the strong interactions with the amino acids in the binding pocket, whereas the left fragment forms less important interactions and is aligned worse. (**F**) Correlation between pIC_50_ values and MM–GBSA binding free energies of UCH-L1 inhibitors docked to the target protein (PDB ID: 4jkj, chain B) using Glide SP.

In the latter phase, we validated the ability of the docking procedure to predict binding affinities of potential PLpro inhibitors. We checked the Pearson correlation coefficients between 21 known pIC_50_ values for different inhibitors and values of scoring functions and binding energies. We repeated the calculations for different PLpro structures retrieved from PDB (Supplementary Table S3). This allowed us to find the best PLpro structure for the drug screening, PDB ID: 7jn2, and the best affinity predictors, which were Jain and MM–GBSA. Additionally, we prepared a MLR model, merging both Jain and MM–GBSA into one function. 7jn2 reached Pearson correlation coefficients of 0.73 (*p* < 0.005) for Jain, −0.64 (*p* < 0.005) for MM–GBSA, and 0.82 (*p* < 0.005) for MLR (Figure 5A–C), and obtained a low RMSD value (1.6 Å) in redocking (Figure 5E) and mostly low RMSD values from cross-docking of ligands from other PLpro crystal structures (Supplementary Table S4).

Finally, we evaluated the selected docking procedure’s ability to correctly predict the binding affinities of potential inhibitors. We prepared an additional set of inhibitors with known IC_50_ values for SARS-CoV-2 PLpro, picking representative compounds in terms of various chemical structures and a wide range of IC_50_ values, together with the previously used molecules giving the total of 50 test compounds. We docked them to 7jn2 and scored analogically as described above. This additional validation step confirmed the docking procedure’s suitability for further screening, with Pearson correlation coefficients of 0.71 (*p* < 0.005) for Jain, −0.55 (*p* < 0.005) for MM–GBSA (Supplementary Figure S1), and 0.75 (*p* < 0.005) for MLR (Figure 5D).

#### 2.4.3. UCH-L1 Binding Affinity Estimation

Before the docking of potential PLpro inhibitors to the selected UCH-L1 structure, we checked the validity of bioactivity predictions for 30 compounds with known IC_50_ values against the hydrolase, made by several docking programs. Therefore, we determined the Pearson correlation coefficients between the pIC_50_ values of the docked ligands and their estimated docking scores or MM–GBSA binding free energies.

The strongest linear correlations were obtained between pIC_50_ values and MM–GBSA binding free energies predicted for ligands docked to the target proteins with PDB ID: 2etl using Glide SP (R = −0.62) and 4jkj using both Glide SP (R = −0.61) (Figure 5F) and Glide XP (R = −0.58). We validated the docking protocol by conducting redocking and cross-docking of the only available UCH-L1 cocrystallized ligand (PDB ID: 4dm9). We docked the molecule to all UCH-L1 crystal structures with Glide SP and Glide XP, and calculated the RMSD of the docking poses relative to the native pose. Considering that the docked ligand was a covalently bound inhibitor, the calculated RMSD values were high, with the average of 5.9 Å for redocking and 10.1 Å for cross-docking. Among the poses obtained from cross-docking, the lowest RMSD values were calculated for the ligand docked to the structure with PDB ID: 2len (RMSD = 6.2 Å) and PDB ID: 4jkj, chain B (RMSD = 6.4 Å) using Glide SP in both cases.

Since the difference between the best Pearson correlation coefficients was small and cross-docking to the structure with PDB ID: 4jkj, chain B using Glide SP gave one of the lowest RMSD values, we selected this entry as the target protein to which we conducted the further docking of potential PLpro inhibitors. We used MM–GBSA binding free energies calculations as a measure to estimate their binding affinities to the selected UCH-L1 structure.

### 2.5. Analysis of the Best Scored Compounds

After all main phases of our screening, we obtained 950 potential PLpro inhibitors. Three hundred eighty-seven of those may be treated as potentially selective, meaning that they should potentially bind well to PLpro and weakly to UCH-L1. We selected the 20 best selective compounds according to their calculated pIC_50_ toward PLpro using our MLR model (Table 1, Supplementary Figure S2). Their IC_50_ values come between 159 and 505 nM. This suggests a potentially higher affinity toward PLpro binding site than for up-to-date synthesized inhibitors. For these 20 compounds, we conducted detailed analysis of their binding poses, the protein–ligand interactions they form, as well as their chemical structures.

#### 2.5.1. Chemical Structures and PLpro Binding Modes

**Figure 6:**
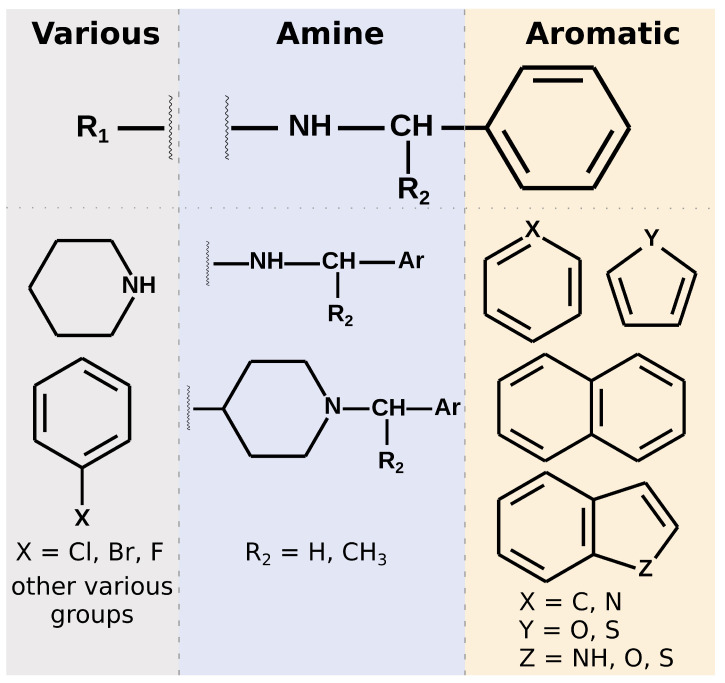
A schematic presentation of the structural features among the 20 potentially selective compounds with the lowest predicted IC_50_ toward PLpro. The compounds’ structure is divided into three main fragments: the right aromatic part, central amine linker, and left, most diversified one. The directions are in agreement with the perspective shown in Figure 1E. The upper panel depicts the simplified structure most often encountered. The bottom part shows possible, most common variations. The fragment of the molecule between wavy lines is also diversified. However, in some cases, a second functional group of the central part, which forms favorable protein–ligand interactions, may be present there.

Most of the selected compounds possess similar structural features compared to the SARS-CoV-2 PLpro noncovalent inhibitors known so far. This is partially an expected outcome considering the structure–ligand-based character of the first step of the screening in LigandScout. However, it also shows that the latter phases in Discovery Studio favor similar compounds, even though there are also slightly different ones among the subset obtained after pharmacophore screening. The analysis of the compounds’ binding modes also shows that, in most cases, the 20 selected molecules adopt poses analogical to those from crystals with complexes of PLpro with noncovalent inhibitors. To simplify the analysis, the compounds’ structures may be divided into three parts. When looking from the perspective as in Figure 1E, BL2 is placed above the inhibitor, the naphthyl group, closer to the fingers subdomain, is situated on the right side, whereas the part near the catalytic triad is located on the left. The right part usually consists of aromatic rings that most often form π–π interactions with Tyr268. The central part comprises crucial hydrophilic groups able to form important hydrogen bonds or salt bridges with nearby residues. Usually, it contains secondary amine groups or amide bonds. The right and central fragments are, in most cases, connected with a methylene group, similarly to the known PLpro inhibitors. The structure of the left part is more diversified and may form not only some hydrogen bonds or salt bridges but also other interactions and, in some cases, only weak ones (Figure 6).

**Figure 7:**
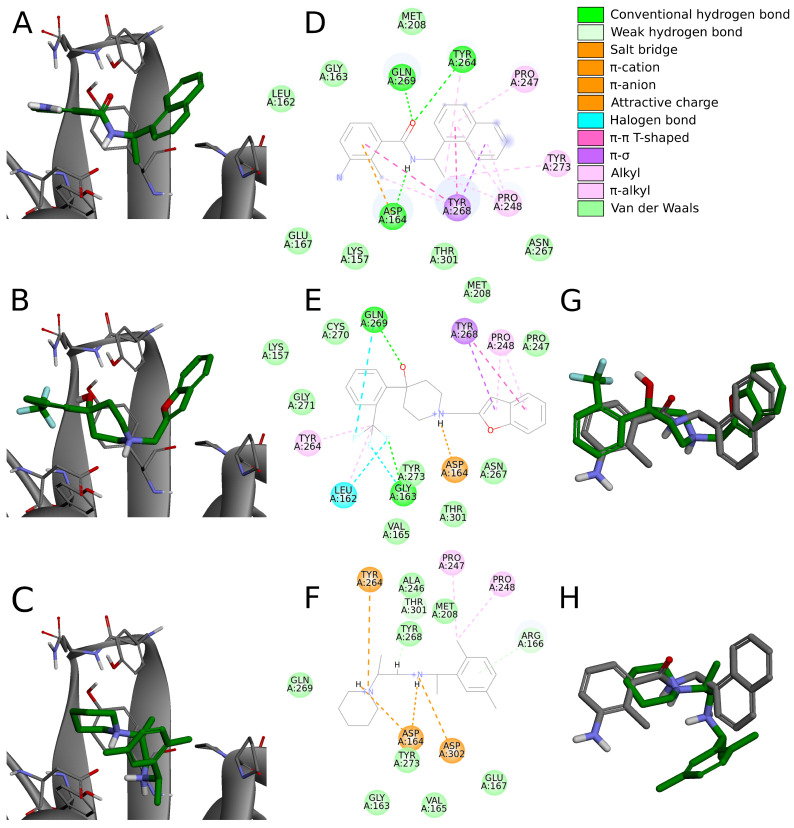
A comparison of binding modes between the inhibitor from the crystal structure (PDB ID: 7jn2) and potential inhibitors from our screening campaign. Panels (**A**–**C**) depict poses of the inhibitor from the crystal structure and potential inhibitors **18** and **3** docked in Discovery Studio to PLpro model based on the aforementioned PDB entry, respectively. Panels (**D**–**F**) show interactions these compounds form with the nearby PLpro residues. (**G**) Binding poses of the inhibitor from crystal structure (gray) and compound **18** (green). (**H**) Binding poses of the inhibitor from crystal structure (gray) and compound **3** (green). Compound **18**, depicted in the middle panels, adopts nearly identical binding pose compared to the crystal inhibitor. Compound **3**, shown in the lower panels, binds in a slightly different manner, utilizing more heavily the left part of the molecule instead of the right aromatic ring.

In detail, the right part of the crystal ligands is built by a naphthalene and forms π–π T-shaped interactions with Tyr268. In our set of the top 20 potential inhibitors, 18 compounds possess an aromatic ring (11) or a polycyclic aromatic scaffold (7) in this part. Among these 18, 12 ligands form interactions with Tyr268, ten of which have a π–π T-shaped character and the latter two are of π-S nature (specific type of the π–lone pair interaction). Interestingly, six of the π–π interactions are formed by polycyclic aromatic structures and only four by single aromatic rings, despite the higher occurrence of the latter. This indicates that in this part of the inhibitor, it is probably preferred to use polycyclic aromatic scaffolds. In the case of the aromatic rings that do not form interactions with Tyr268, they tend to create only other weak interactions instead, whereas the energetically substantially favorable contacts are present in the other parts of those compounds. In those cases, the binding mode of the whole molecule is also slightly different than in crystals (Figure 7A,D) or in our 12 potential inhibitors described above that strongly interact with Tyr268 (Figure 7B,E).

The central part of the noncovalent inhibitors from the crystal structures contains amide groups. In some cases, there may be also present a piperidine connected via the nitrogen atom to the right part of the molecule and via the carbon atom in position 4 to the amide linker in the left (e.g., PDB ID: 7e35). This part of the inhibitor is crucial for the proper steric fit to the narrowest pocket of the binding site, just under the BL2. The functional groups present in the center of the molecule are responsible for the most important interactions with nearby amino acids. These include mainly hydrogen bonds or in some cases salt bridges with Asp164, Tyr264, Tyr268, and Gln269. In the case of the latter two, the interactions are formed by the main chains of these amino acids. Thus, the induced fit effect is of great significance in this context, especially the conformation of the backbone of BL2. Hence, it may be difficult to spot such interactions for specific chemical structures of the inhibitors, specific conformations of PLpro or their combinations. Because we conducted docking to only a single, rigid PLpro structure, it is possible to miss some of the potentially important interactions, as our potential inhibitors slightly vary compared to known, crystallized, noncovalent inhibitors. However, the PLpro model based on PDB ID: 7jn2, that we used in this study, comprises a BL2 conformation with Tyr268 and Gln269 placed similarly to the most of the other crystal, inhibitor-bound structures. The utilization of a representative PLpro structure allows us to model the behavior of this crucial fragment of potential inhibitors in a satisfactory manner.

All of our 20 potential inhibitors in the central part of the molecule contain functional groups that create strong interactions with the binding site. Seventeen compounds possess a secondary amine group, while the other three—tertiary amine in a heterocyclic ring. Additionally, two compounds with secondary amine groups also include concurrent tertiary heterocyclic amines. Three other molecules have a second functional group of another type in the main part—amide, hydroxyl, or ester. All 20 compounds form salt bridges with Asp164. Six potential inhibitors create hydrogen bonds with Tyr273. Seven compounds form π–cation interactions with Tyr264. There are also three molecules with hydrogen bonds with Gln269. Interestingly, these compounds possess more than one functional group in the central part, suggesting that it may be a valid strategy to include in this fragment multiple groups able to create hydrogen bonds or salt bridges.

Both the chemical structures and consequently the interactions formed by the left part of the PLpro inhibitors exhibit a greater variety compared to the rest of the molecule. This fragment of the crystal ligands consists of an aromatic ring or a polycyclic aromatic scaffold. However, it seems to serve little to no purpose itself when it comes to interacting with binding site residues. In some cases there are hydrogen bonds between substituents attached to the aromatic ring and Gln269, Tyr268 or Glu167. Hence, there is room to work on this part of the new potential inhibitors and achieve a structure more suitable for creating a larger number of important interactions with the binding site, compared to the known chemical compounds. In the case of our potential inhibitors, this part is also diversified. Ten of our compounds possess a heterocyclic scaffold, seven of which being a piperidine. Most of these molecules have binding poses placed in such a way to facilitate creating a salt bridge with Asp164, concurrently to a similar interaction of the same amino acid with the central part of the inhibitor. These are usually the compounds that adopt an overall slightly different binding mode than in crystals. They put a bigger emphasis on the interactions of the left part of the inhibitor and do not always form π–π interactions with Tyr268 with the right fragment, which is a characteristic feature of the crystal complexes. Thus, these compounds have the binding poses directed slightly more toward right and their right aromatic part toward bottom, further from the BL2 (Figure 7C,H). Additionally, these potential inhibitors create π–cation interactions with Tyr264 using the left fragment (Figure 7F), contrarily to the compounds with a more crystal-like binding mode that form the same interactions utilizing the central part of the molecule. When it comes to the other chemical constituents in the left fragment, there are three compounds containing a halogenphenyl group. Interestingly, two of them form π–π interactions with Tyr268 here, instead of the right part, being a third, least often observed binding mode. Additionally, these compounds create π–anion interactions with Asp164. Four other potential inhibitors contain various hydrophilic, noncyclic groups in the left fragment, including ester, amide, ether or amine groups. This set of ligands interacts with this region of the binding site in various manners, e.g., via hydrogen bonds with Gln269 or Gly163. Lastly, there are three compounds with only hydrophobic groups in the left part. They do not form any strong interactions using this fragment, owing their possibly high affinity to the favorable contacts in the other regions of the binding site. Similarly to many inhibitors from crystal structures, there may be a possibility to optimize the structure here.

Summarizing the chemical structure of the 20 analyzed potential inhibitors and their binding modes, a few key characteristics should be emphasized. In general, the structures of selected compounds are similar to those from crystal complexes. In the central part, all compounds possess functional groups forming crucial hydrogen bonds or salt bridges, most importantly with Asp164. In the right fragment, the vast majority of the molecules contain an aromatic ring or a polycyclic aromatic scaffold, and the latter seems to be favored. However, only 12 compounds utilize this part of their structure to form interactions with Tyr268, observed in nearly all crystals. A lesser number of potential inhibitors adopt a slightly distinct binding mode, with the lack of the above mentioned contact, and instead with a bigger role of the left fragment. This is especially valid for compounds with a piperidine in the left, as its nitrogen atom forms strong interactions with amino acids in the central pocket of the binding site. Overall, the left fragment of the selected compounds is most diversified both in terms of the chemical structure and interactions. This part seems to be the most promising one for a potential lead optimization.

#### 2.5.2. Detailed Analysis of Compounds with Best Binding Modes

The visual inspection and the analysis of the binding modes of the top scored compounds show that overall they are placed at the binding site similarly to the inhibitors in the crystals. However, only some of them adopt exactly the same binding mode (Figure 7G), while others slightly differ or adopt a wider range of binding modes (Figure 7H). The experimental evidence and knowledge about PLpro inhibitors is still expanding. So far, the in vitro studies have included only a very limited range of structurally relatively similar compounds. Thus, it is difficult to judge whether molecules with different structural features, obtaining in silico slightly varying poses at the binding site, have their predicted binding modes well- or misrepresented. While they are alternative to those from crystals, and as such may be treated as potentially wrong, according to today’s knowledge it is impossible to state that certainly. Hence, if one would want to assess their binding affinity in vitro, it is not an unreasonable choice. Nevertheless, such a direction could be more risky, compared to compounds with binding modes nearly identical to the crystal ones. Therefore, we will focus on such molecules with more conserved poses and will analyze in more detail a few selected, safe proposals.

**Figure 8:**
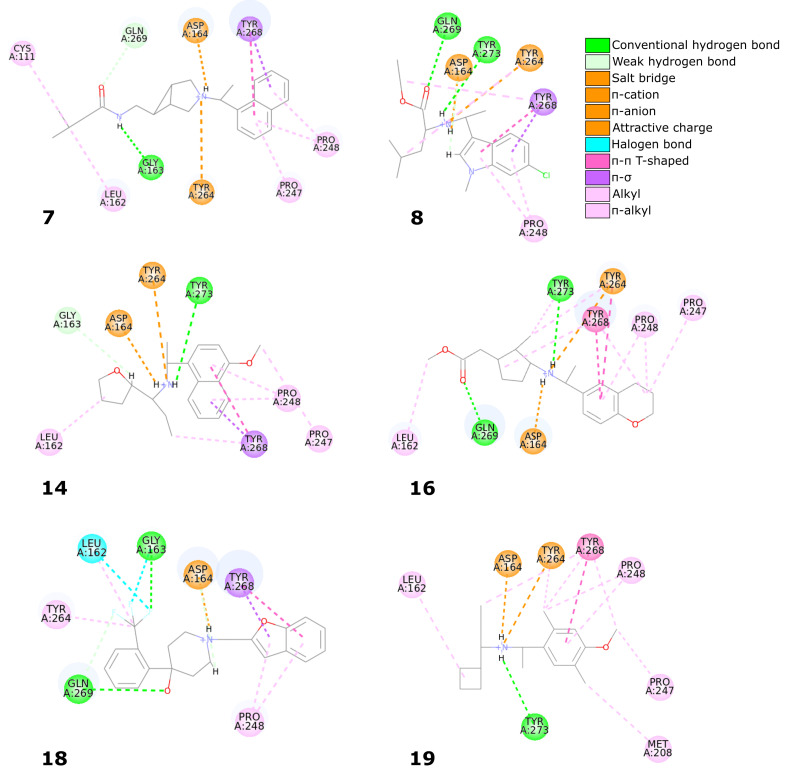
Overview of the six potential SARS-CoV-2 PLpro inhibitors with nearly identical binding mode to the noncovalent inhibitors from the SARS-CoV-2 PLpro crystal structures.

Six compounds from the set of top 20 potential inhibitors (compounds **7**, **8**, **14**, **16**, **18**, and **19**) displayed nearly identical binding mode to the one observed in the SARS-CoV-2 PLpro crystal structures (Table 1, Figure 8). Therefore, we conducted a more detailed analysis of the six molecules based on the PLpro complex with PDB ID: 7jn2.

The biggest similarity to the binding of the cocrystallized ligands was observed for the right and central fragments of the compounds. The right part of all six molecules was composed of one or two fused aromatic rings occupying a hydrophobic cavity hedged by Pro247 and Pro248. This fragment of the selected compounds was buried nearly in the same position as the naphthyl group from the cocrystallized ligand, thereby creating similar interactions. The aromatic rings of the inhibitor from the PDB structure formed π–π T-shaped interaction with Tyr268 and alkyl interactions with Pro247 and Pro248. The first one played the main role in stabilizing the right part of the compound and was observed in all complexes with the six potential inhibitors. The latter interactions were maintained in most cases.

Compounds **7** and **14** showed the biggest similarity in binding of the right part of the molecules. It was due to the fact that these were the only compounds composed of a naphthalene (compound **7**) or its derivative (compound **14**). The substituent in the naphthyl group of the latter compound had no significant impact on its binding beside the additional alkyl interaction with Pro247. The right fragment of compounds **18** and **16** showed a slightly bigger shift from the naphthyl group of the ligand from the crystal structure than the other four potential inhibitors. The benzofuran rings of the compound **18** were more shifted toward the BL2, causing the loss of an alkyl interaction with Pro247, observed in the rest of the complexes. The benzene ring from the chromane forming the right part of the compound **16** was accommodated higher than one of the naphthalene rings from the cocrystallized inhibitor, enabling the molecule to form π–π T-shaped interactions not only with Tyr268 but also with Tyr264.

The central fragment of the inhibitor from the crystal structure consisted of an amide group, which was stabilized by the hydrogen bonds formed with Gln269, Tyr264, and Asp164. Furthermore, multiple alkyl interactions were established between the target protein and a methylene group connecting the right and central part of the ligand. Four out of six potential inhibitors (compounds **8**, **14**, **16**, and **19**) possessed an acyclic secondary amine group in the central part of the molecule that, similarly to the crystal structure, was connected to the right part with a methylene group. All compounds were stabilized by the salt bridges formed between the amine nitrogen and Asp164, which were analogous to the hydrogen bond established in the crystal complex. Although the four compounds lacked the oxygen atom, which was a hydrogen bond acceptor in the crystal structure, the interaction with Tyr264 was still established in the form of the π–cation interaction. We observed that the amine nitrogen atoms from the central part of these four molecules were positioned deeper in the binding site than the amide nitrogen from the cocrystallized ligand. It allowed the central fragment of these potential inhibitors to be additionally stabilized by the hydrogen bond formed with Tyr273.

Unlike the previous four molecules, the central fragment of the compounds **7** and **18** was composed of a tertiary heterocyclic amine. Although both molecules also possessed a methylene group connecting their right and central part, the interactions formed by these two compounds were slightly different. Similarly to the crystal ligand, compound **7** formed the salt bridge and π–cation interactions with Asp164 and Tyr264, respectively. Compound **18** established an additional interaction. The central part of the molecule consisted of a 4-hydroxypiperidine ring. After superimposing the complex with compound **18** onto the crystal structure, we observed that the oxygen atoms from both ligands were localized in the similar position, allowing the potential inhibitor to establish an additional hydrogen bond with Gln269, apart from the salt bridge formed with Asp164.

The left fragment was the most diverse among the compounds. The ligand from the crystal structure possessed the 2-amine-1-methylphenyl group in its left part, which was mainly stabilized by the π–π T-shaped interaction with Tyr268 and π–anion with Asp164. The left fragment of potential inhibitors was composed of various groups. However, not only the chemical properties affected the binding of the compounds in the left part of the PLpro binding site, but also their size. Compound **7** was longer than the cocrystallized ligand and did not bind in the bent conformation around the BL2. Therefore, the molecule established a hydrogen bond with Gly163 and alkyl interactions with Cys111 and Leu162. The other potential inhibitors were of similar length to the ligand from the crystal structure. The left terminal part of the compounds **8** and **16** consisted of the ethoxycarbonyl and methoxycarbonyl groups, respectively. Both compounds were stabilized by a hydrogen bond formed between the carbonyl oxygen and Gln269. In terms of the left fragment, compound **18** stood out the most from the rest of potential inhibitors. The molecule possessed the trifluoromethylphenyl group, which was accommodated in the similar position as the benzene ring from the cocrystallized inhibitor. The compound established multiple strong interactions with the target protein—four halogen bonds between the fluorine atoms and the residues Gln269, Leu162, Gly163, and also a hydrogen bond with the latter. All molecules were additionally stabilized by a few alkyl interactions.

In conclusion, the six potential PLpro inhibitors displayed similar binding mode to the noncovalent ligands from the crystal structures and in some cases they also formed additional interactions, which is one of the main factors of their potentially very high binding affinity. Most interactions stabilizing the naphthyl group of the cocrystallized inhibitor were maintained in the analogous groups of all six compounds. The central fragment of the analyzed molecules formed several interactions similar to the ones observed in the compared crystal structure, but it also established some additional ones, in most cases strong hydrogen bonds. The fragment located the closest to the catalytic triad was the most diverse among the compounds. Although in some cases this part of the molecules formed strong interactions with the PLpro binding site, several potential inhibitors were only stabilized by the alkyl interactions. Therefore, optimization of this fragment of the compounds may lead to the enhancement of binding affinity.

#### 2.5.3. Binding Modes from the Perspective of the Protein

**Figure 9:**
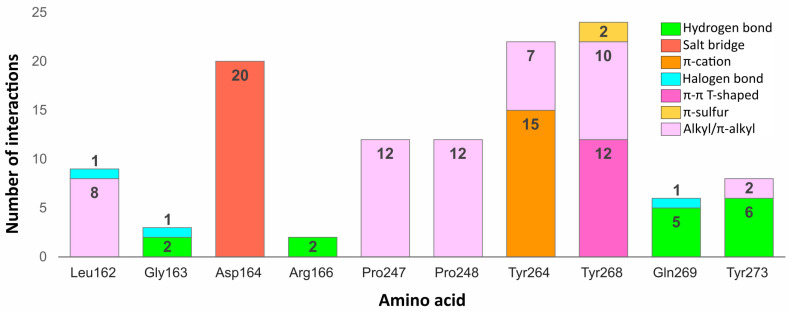
Bar graph showing selected amino acids from the SARS-CoV-2 PLpro binding site (PDB ID: 7jn2) and the number of interactions formed by each with top 20 potential inhibitors. The numbers included in the graph represent the number of compounds with which an amino acid has formed a given type of interaction.

Some residues in the PLpro binding site were especially important in forming interactions with the potential inhibitors (Figure 9). Two of the key amino acids were Tyr268 and Gln269 placed in the flexible BL2. The first residue stabilized the ligand poses by forming interactions with the aromatic rings mainly located in a hydrophobic cavity hedged by Pro248, Pro247, and Met208. The side chain of Tyr268 established π–π T-shaped interactions with 12 ligands and π-S with two ligands from the set of top 20 potential inhibitors. Gln269, however, was involved in stabilizing the central part and the other end of the compounds by creating hydrogen bonds with five potential inhibitors, three of which interacted with the backbone and two with the side chain of the amino acid. The interactions formed between the molecules and the residues Tyr268 and Gln269 were similar to those observed in most inhibitor-bound SARS-CoV-2 PLpro crystal structures.

The flexible BL2 may adopt various conformations among different structures depending on the interacting compound. However, due to the fact that most cocrystallized SARS-CoV-2 PLpro inhibitors adopt nearly identical binding mode, the BL2 of the analyzed crystals, especially the backbone and side chains of two important binding residues—Tyr268 and Gln269, show great conformational similarities. One of the few examples, where the BL2 is differently arranged is the structure with PDB ID: 7e35, bound to the compound with a different chemical structure (derivative of rac3j) and a binding mode compared to the rest of the crystal ligands. In this case, the backbone of Tyr268 and Gln269, and the side chain of the latter residue adopt a more open conformation due to the induced fit mechanism. This might result in a decreased formation of some of the aforementioned interactions, namely the hydrogen bonds established between certain potential PLpro inhibitors and the backbone and side chain of Gln269. However, the carbonyl oxygen of Tyr268 in the structure with PDB ID: 7e35 is shifted toward the binding site, which may potentially induce the formation of new hydrogen bonds. Additionally, considering that the aromatic side chain of Tyr268, engaged in forming important π–π contacts, adopts an almost identical conformation in all of the analyzed inhibitor-bound SARS-CoV-2 PLpro crystals, it is highly probable that the overall interaction profile would be maintained in various structures, regardless of their BL2 arrangement.

Another residue, which played an important role in stabilizing both the cocrystallized inhibitors and selected compounds was Asp164. The amino acid occupying the central part of the PLpro binding site, formed salt bridges with all 20 potential inhibitors, which were analogous to the hydrogen bonds observed in the SARS-CoV-2 PLpro crystal complexes.

Two tyrosine residues located close to the BL2 were also engaged in forming relevant interactions with selected compounds. Tyr264 created π-cation interactions with the protonated nitrogen atoms from the central or left fragment of 15 molecules. The second tyrosine residue—Tyr273, formed hydrogen bonds with amine groups of six compounds from the set. Although the latter amino acid did not form relevant interactions in the PLpro crystal complexes, it turned out to be an important residue for binding some of the top 20 potential inhibitors.

Apart from the mentioned tyrosine, there were a few more amino acids, which formed relevant interactions with the PLpro potential inhibitors, despite not playing any important role in stabilizing the cocrystallized ligands. These were mainly residues occupying the left part of the binding site, which was localized closer to the catalytic triad. Arg166 and Gly163 were both engaged in binding two compounds by forming the hydrogen bonds with each. In one of the newly obtained complexes, we observed the appearance of a type of interaction, which was not present in the crystal structures, namely the halogen bond. The interaction was formed with Leu162, Gly163, and previously analyzed Gln269 from the BL2.

We noticed some similarities between the weak interactions established in the PLpro crystal structures and the complexes obtained from the virtual screening workflow. The right part of the molecules occupied a hydrophobic cavity formed by Pro248, Pro247, and Met208. Sixteen out of 20 analyzed ligands were engaged in forming alkyl or π-alkyl interactions with one or both prolines from the pocket, which resembled the binding of the naphthyl group in the cocrystallized inhibitors. The amino acids from the central part of the PLpro binding site—Tyr264, Tyr268, and Tyr273 also showed an analogous tendency of forming π-alkyl interactions. Leu162, which was one of the most often encountered residues stabilizing the left part of potential inhibitors with alkyl interactions, was not involved in binding the ligands from the crystal structures.

In conclusion, most of the interactions formed between potential inhibitors and the target protein were analogous to those observed in the SARS-CoV-2 PLpro crystal structures. However, some amino acids, which did not seem to be relevant in binding the cocrystallized inhibitors, turned out to be engaged in forming important interactions in the complexes obtained as a result of our research. Thus, there is a high possibility that more PLpro binding site residues could be engaged in forming relevant interactions than it may appear from the analysis of the crystal structures. Therefore, it is feasible to design new, potent PLpro inhibitors, which would interact with a greater number of amino acids than the cocrystallized compounds reported so far.

#### 2.5.4. UCH-L1 Binding Modes

We examined the protein–ligand interactions of UCH-L1 using the top 20 potential PLpro inhibitors from the screening. For those compounds, obtained docking scores suggest a low probability of binding to UCH-L1. To support this result, we analyzed the complexes obtained from docking to the UCH-L1 from the PDB ID: 4jkj crystal structure.

We compared protein–ligand interactions to the 4dm9 crystal structure as a reference, as it is the only available UCH-L1 PDB structure with an inhibitor. 4dm9 was cocrystalized with the covalent inhibitor Z-VAE(OMe)-FMK (benzyloxycarbonyl-Val-Ala-Glu(γ-methoxy)-fluoromethylketone) [39]. The compound irreversibly modifies UCH-L1 by binding covalently to Cys90, which forms, along with His161 and Asp176, the catalytic triad [45].

Despite that 4dm9 may not be an ideal reference structure, due to the different ligand binding type, the similarities are sufficient to provide relevant comparison. The described comparative interaction analysis of the covalently bonded Z-VAE(OMe)-FMK and other noncovalently bonded compounds constitutes a reliable foundation for the prediction of potential toxicity.

**Figure 10:**
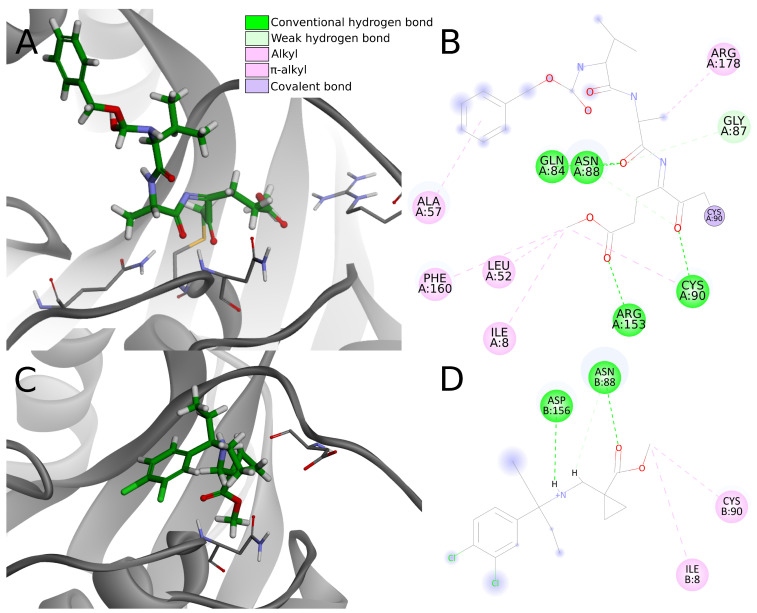
A comparison of ligand–protein interactions between the crystal structure (PDB ID: 4dm9) and the potential inhibitor from the screening campaign. The upper panels show a 3D view of ligand binding mode (**A**) and 2D interaction diagram (**B**) for Z-VAE(OMe)-FMK covalent inhibitor. Panel (**C**) shows biding mode of compound **9** from screening. In panels (**A**,**C**) the residues that create hydrogen bonds with inhibitors were shown in a sticks representation. Panel (**D**) shows a 2D interaction diagram for potential PLpro inhibitor, compound **9**. Chosen compound creates visibly fewer interactions, including hydrogen bonds, than the inhibitor from the crystal structure.

In the analysis of the 4dm9 crystal structure, apart from covalent bonds, noncovalent interactions can be seen: hydrogen bonds with Gln84, Asn88, Cys90, and Arg153, weak carbon–hydrogen bonds with Arg153, alkyl hydrophobic interactions with Ile8, Leu52, Cys90, and Arg178 and π–alkyl hydrophobic interactions with Ala57 and Phe160 (Figure 10A,B).

Considering interactions appearing in the 4dm9 crystal, among the top 20 compounds docked to 4jkj three create hydrogen bonds with Gln84, eight with Asn88, two with Cys90 and none with Arg153. In terms of hydrophobic interactions, eight compounds create alkyl bonds with Ile8 and 11 with Cys90. Three of the chosen ligands show none of the interactions indicated for the 4dm9 crystal structure and another six—only one of indicated interaction.

The chosen compounds also create other interactions not occurring in the reference crystal structure. Seventeen out of 20 compounds form hydrogen bonds with Asp156 and seven with Val158. Nine compounds create salt bridges with Asp155 and 16 compounds form an attractive charge interaction with Asp155 or Asp156.

As hydrogen bonds are strong interactions and are considered as the most important ligand binding factor, summing them up enables clear comparison. Five compounds (numbers **1**, **2**, **5**, **15**, and **18**) create one hydrogen bond, nine (**3**, **4**, **6**, **7**, **9**, **11**, **13**, **19**, **20**) have two hydrogen bonds, five (**8**, **10**, **12**, **14**, **17**)—three hydrogen bonds and only one, compound **16**, creates four. As it can be seen, the vast majority of the compounds (19 out of 20) create fewer hydrogen bonds than are present in the reference crystal structure. Four of the compounds have unfavorable interactions, including compound **16** (donor-donor unfavorable interaction with Ans88), which had been pointed out before as having a greater number of hydrogen bonds.

Differences in binding strength become more pronounced considering the fact that in Z-VAE(OMe)-FMK the strong covalent bond provides stable ligand binding in the active site. Noncovalent interactions have only a supportive function, so there are fewer of them. Compounds chosen in the conducted screening are designed as noncovalent inhibitors, so they bind to the protein thanks to multiple noncovalent, relatively weaker interactions.

Having less or the same number of strong noncovalent interactions as in 4dm9, the top 20 potential PLpro inhibitors have very little chance to create stable binding to UCH-L1. Apart from the lack of a covalent bond, most compounds create less than three conventional hydrogen bonds, one or two electrostatic interactions, less than three hydrophobic interactions and multiple weak interactions such as carbon hydrogen bonds (Figure 10C,D). Although the overall number of interactions is greater in some cases, this does not necessary imply stronger binding affinity. Lacking a covalent bond and abundant, strong noncovalent interactions, compounds are rather unable to create stable binding to UCH-L1.

Even though some of the compounds create hydrogen bonds and salt bridges with UCH-L1 residues, it is unlikely that they are sufficient to induce strong ligand binding. Together with the docking score results, protein–ligand interaction analysis suggests that the compounds chosen in the screening have a low probability of exhibiting toxicity due to inhibiting UCH-L1.

#### 2.5.5. Toxicity Estimation

For top 20 potential PLpro inhibitors from screening, we conducted an approximative toxicity prediction. Based on the results, only three compounds exhibit mutagenicity. Thirteen compounds are probably developmental toxicants. Most compounds show a relatively high LD_50_ value. In general, the top 20 compounds do not appear to be particularly toxic, however most of them may not be suitable for pregnant women and children (Table 2).

## 3. Materials and Methods

### 3.1. Ligands Database for Screening

In this project we used the ENAMINE REAL database, containing 15,547,092 drug-like, diverse compounds on the date of acquisition (13 July 2020). These compounds possess drug-like properties, fulfilling the Lipinski [53] and Veber [54] rules, including molecular weight (MW) ≤ 500 g/mol, ClogP ≤ 5, hydrogen bond donors (HBD) ≤ 5, hydrogen bond acceptors (HBA) ≤ 10, rotating bonds ≤ 10, topological polar surface area (TPSA) ≤ 140 Å^2^, and lack of PAINS. Additionally, the library contains no compounds with Tanimoto similarity above 0.6 in relation to other molecules within the set.

### 3.2. Pharmacophore Screening

For creation of pharmacophores and initial screening of drugs we used LigandScout 4.4.5 [55]. Initially, we designed a set of pharmacophores and later tested their ability to detect active compounds. We created pharmacophores from protein–ligand complexes retrieved from PDB (PDB IDs: 6wuu (all chains), 6wx4 [30], 7jir, 7jit, 7jiv, 7jiw, 7jn2, 4ovz (chain A), and 3mj5). In 6wuu and 6wx4, we manually separated covalently bonded peptide ligands from Cys111 in BIOVIA Discovery Studio and fixed peptide bonds before creating pharmacophores. 7jir, 7jit, and 7jiv structures had a C111S mutation so we changed their Ser111 back into cysteine in Discovery Studio. The initial pharmacophores were then merged by reference points in different combinations to make them less specific toward one type of ligand.

Pharmacophores were tested for their ability to pick up potent inhibitors out of a set of decoys by screening and comparing the enrichment factor 1% and the Pharmacophore Fit Score for the active ligands. We chose high-affinity inhibitors out of compounds with the best IC_50_ values from in vitro studies: GRL0617, rac3j_R, rac3k_R, rac5c_R (active R enantiomers of rac3j, rac3k, and rac5c, respectively) and peptide inhibitors from 6wuu and 6wx4 (VIR250 and VIR251, respectively). As a decoy database we used a preprepared drug-like ligand decoys set from Schrödinger containing 1000 compounds. Both databases were prepared for screening using the idbgen function with the high-throughput iCon Fast option and with max conformations: 100. The screening consisted of fitting multiple ligand conformations into a rigid pharmacophore. We chose the Get Best Matching Conformations retrieval mode and used different values of Max. Number of Omitted Features to find the optimal screening parameters.

The validation allowed us to find a pharmacophore that was used for the subsequent drug screening. The chosen pharmacophore was created from protein–ligand complexes PDB IDs: 7jiw, 7jn2, 4ovz (chain A), and 6wuu (chain C) to ensure that every inhibitor structural type was taken into account with as few initial pharmacophores as possible. We manually modified the pharmacophore and changed two default descriptors (hydrophobic spheres), present in the initial naphthyl group of 7jiw, 7jn2, and 4ovz, to aromatic rings in order to emphasize the importance and directionality of π–π interactions between inhibitors and Tyr268. We removed two H-bond acceptor descriptors in the central part of the 7jiw initial pharmacophore, as they contributed to high bias toward GRL0617 type of ligands. Based on the validation results we decided that the Max. Number of Omitted Features of 14 was optimal for this drug screening since 110 hits were reported with five being true positive (GRL0617 placed first, VIR251—2nd, rac3j_R—3rd, rac3k_R—4th, rac5c_R—14th). Performance of the pharmacophore for different numbers of omitted features is presented in Supplementary Table S5. In order to confirm the selected pharmacophore’s ability to identify potent PLpro inhibitors, we performed an additional test screening. The step was conducted on the same set of decoys and the bigger set of active compounds composed of 20 SARS-CoV-2 PLpro inhibitors with IC_50_ values below 1 μM (Supplementary Table S6). All other parameters were kept unchanged. For the ENAMINE library preparation and the subsequent drug screening we used the same functions and options as during the validation.

### 3.3. Docking and Binding Energy Calculations—PLpro

For docking to PLpro and calculations of binding energy, we used BIOVIA Discovery Studio v20.1.0.19295 [56]. The structures retrieved from PDB were prepared using the Prepare proteins protocol with pH set to 7.4 and the CHARMm forcefield. We used spherical gridboxes with a radius of 15 Å that were created around a ligand if the PLpro complexes featured one, or around the PDB ID: 3e9s ligand after superimposing the proteins by Cα atoms. We prepared the potential inhibitors using the Prepare ligands protocol and changed their ionization for pH 7.5 ± 1 without generating tautomers and isomers. Afterward, we employed the CDOCKER protocol, a tool performing grid-based docking to a rigid protein with ligand conformational sampling utilizing molecular dynamics. We retrieved only the best pose for each ligand by setting the option top hits to 1. After docking, we assessed the obtained poses by scoring functions in the Score ligand poses protocol and utilized the Calculate binding energies protocol with generalized Born implicit solvent model (MM–GBSA). Afterward, we created a MLR model by merging the Jain scoring function and the MM–GBSA binding energy. For this purpose, we utilized Statistica 13.1 software [57].

Initially, we validated the ability of the software to predict the correct inhibitor poses by employing the redocking and cross-docking techniques. We retrieved various PLpro structures (PDB IDs: 7jir, 7jit, 7jiv, 7jiw, 6wuu (all chains), 6wx4, 6w9c (chain A), 6xa9 (chain A), 7jn2, 7jrn (all chains), 7cjm, 7cmd, and 7cjd (all chains)) and redocked their original inhibitors as well as cross-docked inhibitors from other PLpro complexes. Additionally, we docked a few ligands found in complexes of SARS-CoV PLpro (PDB IDs: 3mj5, 4ovz, and 4ow0). The obtained protein–ligand complexes were superimposed by Cα atoms on the original PLpro complexes with the corresponding ligands in order to calculate ligand heavy-atom RMSD values. Based on the validation results we chose the PLpro structures with the lowest cross-docking RMSD values and used them to test the ability of the docking procedure to predict binding affinities of inhibitors. Two of the chosen structures (PDB IDs: 7jiv and 7jit) had a C111S mutation so we created additional structures where the serine residue was mutated back into cysteine. We created a set of 25 PLpro inhibitors with known IC_50_ values (Supplementary Table S7). After docking them to the chosen PLpro structures, we assessed the obtained poses by scoring functions (CDOCKER Energy, CDOCKER Interaction Energy, LigScore1, LigScore2, PLP1, PLP2, Jain, PMF, PMF04) and MM–GBSA calculations. Subsequently, we checked Pearson correlation coefficients and the corresponding *p*-values between pIC_50_s and the values obtained from scoring functions and binding energy calculations. If an inhibitor had its IC_50_ measured for a racemate, we took an average of scoring functions and binding energies calculated for both enantiomers. Through this validation, we found the best PLpro structure, which was used for the subsequent drug screening. Additionally, we prepared a MLR model for this structure and the best-performing scoring function (Jain) and MM–GBSA. To validate the model, we examined *p*-values for the model itself as well as for all the terms of the equation. All *p*-values came below 0.01. Due to the simplicity of the model and to the occurrence of only two independent variables, we did not conduct cross-validation.

We conducted an additional step of validation in order to confirm the best-performing docking procedure’s (PDB ID: 7jn2, Jain, and MM–GBSA) ability to correctly predict potential inhibitors’ binding poses and affinities toward PLpro. First, we docked inhibitors cocrystallized with PLpro structures available in PDB that we did not utilize in the main cross-docking validation (PDB IDs: 7koj, 7kok, 7lbr, 7lbs, 7llf, 7llz, 7los, and 7e35). We superimposed the complexes and calculated the ligand’s heavy atom RMSD analogically as described above. Next, we created an additional set of PLpro inhibitors with known IC_50_ values (Supplementary Table S8). We docked them to 7jn2 and conducted analogical scoring and binding energy calculations as described above. We determined Pearson correlation coefficients and the corresponding *p*-values between the experimental pIC_50_ values and the values of Jain function, MM–GBSA binding energies, and pIC_50_ values estimated using the MLR model for the combined two sets of test ligands.

In the screening part, we prepared the potential inhibitors and docked them using the same procedure as during validation, and then assessed the obtained poses by the Jain scoring function and MM–GBSA calculations.

### 3.4. Docking and Binding Energy Calculations—UCH-L1

In order to check the validity of bioactivity predictions made by docking programs (CDOCKER and Glide), we checked how they perform on a set of 30 compounds with known in vitro activity against UCH-L1 (IC_50_ value). After comparing both programs, we noticed that significantly better results were obtained from docking in Glide. Therefore, the following steps refer to the workflow employed in the latter.

First, we generated 3D conformations of the selected compounds in Maestro 2017-1 software [58]. The set of compounds was prepared using the LigPrep protocol; we generated possible protonation states in the pH range 7.0 ± 2.0 using Epik. We retrieved the total of eight crystal structures of UCH-L1 from the PDB, among which one structure was in complex with a covalent ligand (PDB ID: 4dm9), three structures were in ubiquitin-vinyl methyl ester (UbVMe) bound forms (PDB IDs: 3kw5, 3kvf, and 3ifw), and four structures were in ligand-free forms (PDB IDs: 3irt, 2len, 4jkj, and 2etl).

The structures were prepared in Maestro 2017-1 using Protein Preparation Wizard. After removing water molecules, we added hydrogen atoms and generated probable protonation states using Epik in the pH range 7.0 ± 2.0. We optimized the H-bond assignment using PROPKA and minimized the structures in the OPLS3 force field. If a PDB entry consisted of multiple chains, we conducted the docking to each of them.

Defining the grid coordinates depended on whether a structure contained a cocrystallized ligand. We determined the center of the box for ligand-bound forms according to the centroid of the defined ligand molecule. The grids for ligand-free and UbVMe bound structures were generated based on the amino acid residues involved in the interactions with the crystal bound ligand of the PDB entry 4dm9. We established the center coordinates by indicating the following residues: Met6, Gln84, Asn88, Ser89, Cys90, Arg153, Asn159, Phe160, and Arg178. The size of the receptor grid was set at default 20 Å. The set of selected compounds was docked to the UCH-L1 structures with Glide Standard Precision (SP) and Glide Extra Precision (XP) using the default settings.

Before validating the bioactivity estimations made for the set of 30 selected compounds, we conducted redocking and cross-docking of the only available UCH-L1 cocrystallized ligand (PDB ID: 4dm9). We prepared the inhibitor in Maestro 2017-1 by initially removing the covalent bond between the compound and the target protein, adding a missing fluorine atom and conducting geometry minimization using the Minimize Selected Atoms feature. The docking was then conducted to all aforementioned UCH-L1 crystal structures using Glide SP and Glide XP. In order to validate the process, we calculated RMSD between the heavy atoms of native and docking poses, after prior superposition of Cα atoms from the obtained complexes onto the crystal structure (PDB ID: 4dm9).

To check the validity of bioactivity predictions made by Glide, we docked 30 compounds with known in vitro activity against UCH-L1 (Supplementary Table S9) and calculated the Pearson correlation coefficient (R) between their pIC_50_ values and their estimated docking scores or MM–GBSA binding free energies. Therefore, we generated the best pose for each docked compound and predicted its binding affinity using the DockingScore scoring function and conducting MM–GBSA binding free energy calculations using Prime (ΔG_bind_ [kcal/mol]).

### 3.5. Toxicity Prediction

We utilized TEST 4.2.1 software to estimate selected toxicological properties of the top 20 potential PLpro inhibitors. We calculated Ames mutagenicity, developmental toxicity, and oral rat LD_50_. For all calculations, we used consensus method, which averages the predicted toxicities from all quantitative structure–activity relationship (QSAR) submethods.

### 3.6. Database

The database page (https://plpro-inhibitors.cent.uw.edu.pl) was generated using the csvtotable Python package. The images of chemical compounds were downloaded from the ChemDB Chemoinformatics Portal [59].

## 4. Conclusions

Because of the scale and significance of the COVID-19 pandemic, it is of utmost importance to design specific anti-SARS-CoV-2 drugs. One of the most suitable molecular targets for this task is the papain-like protease. Here, we selected the best potential candidates for potent and selective PLpro inhibitors. For this purpose, we established a multistep virtual screening workflow. To the best of our knowledge, it is the most meticulously validated and probably the most accurate procedure for computational prediction of SARS-CoV-2 PLpro inhibitors to this date. In our approach, we utilized three main drug design programs, with several, diverse ways of the evaluation of the potential PLpro inhibitors. We considered potential toxicity of the drug candidates at the early stages of the design. In this manuscript, we put emphasis on the most important structural analog of SARS-CoV-2 PLpro in the human organism—UCH-L1. Additionally, we also roughly estimated other toxicological parameters.

The comprehensive analysis of the results led to identification of important structural and binding patterns of potential inhibitors. These properties are in agreement with those that may be drawn from the overview of the PLpro-inhibitor crystal structures. Moreover, our results provide additional information on binding modes of potential PLpro inhibitors as well as this enzyme’s amino acids that may be involved in forming significant interactions with drug-like compounds.

The analysis of the chemical structure of our top potential hits shows that their preferred scaffold is in general similar to the known noncovalent inhibitors. However, the present differences allow to potentially encounter a slightly distinct, better compound. The successful drug candidate should consist of an aromatic moiety on one side, a central fragment containing functional groups able to create hydrogen bonds or salt bridges, and a less defined moiety on the other side. The aromatic part should comprise preferably a bicyclic scaffold. We shown that the central part, instead of containing amide group, may include an amine group and it may be beneficial to add additional ones, i.e., hydroxyl groups. The last fragment of the potential PLpro inhibitors has the least number of structural requirements. Thus, this part of the molecule is a valuable space for future lead optimization.

The analysis of the binding modes of selected potential PLpro inhibitors led also to important conclusions from the perspective of the protein itself as well as amino acids crucial for small molecule binding, that may be also exploited while designing new drugs. Our results show that there are several residues that stand out in the frequency of forming relevant interactions with noncovalent ligands. The most significant ones include Asp164, Tyr264, Tyr268, Pro247, and Pro248. Moreover, we encountered amino acids that are not relevant for binding inhibitors from the crystal complexes but may be important for binding the compounds with different chemical structures. The most prominent ones are Tyr273, being able to create hydrogen bonds, and Leu162, forming multiple hydrophobic interactions.

Herein, we identified 950 potential SARS-CoV-2 PLpro inhibitors. Among these, 387 are potentially selective, with low predicted affinity toward human UCH-L1. The predicted IC_50_ values of the 20 top scored of these compounds come between 159 and 505 nM. Based on their detailed analysis, we proposed six of them as very promising candidates for future in vitro evaluation. These compounds exhibit similar binding mode to the noncovalent inhibitors from the crystal structures and also create additional interactions, which is one of the key factors of their potentially high binding affinity. However, all the 950 potential PLpro inhibitors are also worth taking into account for future experimental evaluation. Thus, we prepared an open-access database containing all of them, with results of our in silico predictions. Such a database may be very useful for other scientific groups and may potentially help to fight the COVID-19 pandemics.

## Figures and Tables

**Table 1:** Twenty compounds chosen from the 387 potential selective PLpro inhibitors, with the lowest pIC_50_ values based on the MLR model estimation. ID numbers in the first column are given to these compounds according to the increasing values from the second column—the IC_50_ toward PLpro predicted using our MLR model. The next two columns show values of the MM–GBSA protein–ligand binding energies and Jain scoring function obtained after docking to PDB ID: 7jn2 PLpro model using Discovery Studio CDOCKER. The fifth column shows the values of MM–GBSA protein–ligand binding energies calculated after docking to UCH-L1 model based on PDB ID: 4jkj in Maestro Glide. As the binding energies toward these two proteins were estimated with different software, force fields, and settings, their values are not comparable. The last column depicts the results of our visual inspection of the binding poses obtained after docking to PLpro in Discovery Studio. “+++” indicates a binding pose nearly identical to the crystal (PDB ID: 7jn2), while “−” an entirely different pose.

ID	Predicted IC_50_ (nM)	Binding Energy—PLpro (kcal/mol)	Jain—PLpro	Binding Energy—UCH-L1 (kcal/mol)	Visual Inspection PLpro
1	159	−13.4	10.7	−29.6	++
2	226	−19.2	9.2	−29.3	+
3	227	−16.9	9.5	−19.7	+
4	248	−19.2	9.0	−28.4	−
5	270	−18.1	9.0	−24.2	+
6	287	−17.6	9.0	−26.6	++
7	303	−23.0	8.2	−27.2	+++
8	324	−20.4	8.4	−23.7	+++
9	359	−17.6	8.6	−29.7	−
10	370	−25.0	7.5	−30.0	++
11	383	−12.0	9.3	−20.6	+
12	385	−18.2	8.4	−28.0	+
13	420	−13.9	8.8	−16.0	++
14	432	−19.6	8.0	−26.1	+++
15	433	−17.7	8.2	−25.0	++
16	446	−13.5	8.8	−29.6	+++
17	476	−15.0	8.4	−20.4	++
18	483	−18.7	7.9	−27.1	+++
19	498	−17.8	8.0	−21.1	+++
20	505	−19.7	7.7	−29.8	+

**Table 2:** Toxicity prediction for top 20 compounds from screening campaign. The “+” sign indicates positive result in mutagenicity or developmental toxicity prediction, “−” was used for nonmutagenic and nontoxic compounds.

	Mutagenicity		Developmental Toxicity		Oral rat LD_50_
ID	Predicted Value	Mutagenicity	Predicted Value	Developmental Toxicity	Predicted Value (mg/kg)
1	0.85	+	0.80	+	1036.8
2	0.13	−	0.23	−	742.9
3	0.23	−	0.26	−	748.1
4	0.27	−	0.87	+	356.6
5	0.13	−	0.51	+	1133.2
6	0.12	−	0.64	+	279.6
7	0.56	+	0.83	+	485.1
8	0.48	−	0.50	−	2234.4
9	0.05	−	0.85	+	696.8
10	0.10	−	0.68	+	180.2
11	0.30	−	0.36	−	382.7
12	0.55	+	0.26	−	908.8
13	0.33	−	0.88	+	2121.2
14	0.41	−	0.44	−	579.3
15	0.08	−	0.55	+	988.2
16	0.03	−	0.72	+	503.5
17	0.35	−	0.38	−	435.4
18	0.37	−	0.76	+	129.2
19	0.24	−	0.60	+	1023.1
20	0.35	−	0.58	+	705.1

## Data Availability

The results for the most promising, potential SARS-CoV-2 PLpro inhibitors are deposited at https://plpro-inhibitors.cent.uw.edu.pl.

## Supplementary Materials

The following are available online at https://www.mdpi.com/article/10.3390/ijms22083957/s1, Figure S1: Correlation between values of scoring functions and binding energies, and pIC_50_ values of the extended set of inhibitors docked to PLpro, Figure S2: Structural formulas of the 20 potentially selective PLpro inhibitors candidates with the lowest predicted IC_50_ values based on the MLR model estimation, Table S1: Summary of SARS-CoV-2 PLpro crystal structures available in the PDB database, Table S2: Cross-docking ligand heavy-atom RMSD [Å] after superposition of proteins by Cα atoms, Table S3: Pearson correlation coefficients between pIC_50_ values of inhibitors and values of scoring functions and MM–GBSA, Table S4: Cross-docking results for 7jn2, Table S5: Performance of the selected pharmacophore for different numbers of omitted features, Table S6: SARS-CoV-2 PLpro inhibitors used for the confirmatory validation of the best pharmacophore, Table S7: PLpro inhibitors used for validation of binding affinity prediction, Table S8: PLpro inhibitors used for additional validation of binding affinity prediction, Table S9: Summary of compounds with known in vitro activity against UCH-L1 used for molecular docking validation conducted in Maestro 2017-1.

## Abbreviations

The following abbreviations are used in this manuscript:

BL2	blocking loop 2
COVID-19	coronavirus disease 2019
DUB	deubiquitinating enzyme
EF	enrichment factor
HBA	hydrogen bond acceptor
HBD	hydrogen bond donor
HCV	hepatitis C virus
HIV	human immunodeficiency virus
IC_50_	half maximal inhibitory concentration
ISG	interferon-stimulated gene
ISG15	interferon-stimulated gene 15
LD_50_	lethal dose, 50%
MERS-CoV	Middle East respiratory syndrome coronavirus
MLR	multiple linear regression
MM–GBSA	molecular mechanics—generalized Born and surface area solvation
Mpro	main protease
MW	molecular weight
PDB	Protein Data Bank
PLpro	papain-like protease
QSAR	quantitative structure–activity relationship
RMSD	root-mean-square deviation
SARS-CoV	severe acute respiratory syndrome coronavirus
SARS-CoV-2	severe acute respiratory syndrome coronavirus 2
SP	standard precision
TEST	Toxicity Estimation Software Tool
TPSA	topological polar surface area
Ub	ubiquitin
Ubl	ubiquitin-like (domain)
UbVMe	ubiquitin-vinyl methyl ester
UCH-L1	ubiquitin carboxy-terminal hydrolase L1
UCH-L3	ubiquitin carboxy-terminal hydrolase L3
WHO	World Health Organization
XP	extra precision

## References

[1] World Health Organization WHO Director-General’s Opening Remarks at the Media Briefing on COVID-19—11 March 2020.

[2] Zhu N., Zhang D., Wang W., Li X., Yang B., Song J., Zhao X., Huang B., Shi W., Lu R. (2020). A novel Coronavirus from Patients with Pneumonia in China, 2019. N. Engl. J. Med..

[3] Chen N., Zhou M., Dong X., Qu J., Gong F., Han Y., Qiu Y., Wang J., Liu Y., Wei Y. (2020). Epidemiological and clinical characteristics of 99 cases of 2019 novel coronavirus pneumonia in Wuhan, China: A descriptive study. Lancet.

[4] Dong E., Du H., Gardner L. (2020). An interactive web-based dashboard to track COVID-19 in real time. Lancet Infect. Dis..

[5] Center for Systems Science and Engineering (CSSE) at Johns Hopkins University (JHU) COVID-19 Dashboard. https://www.arcgis.com/apps/opsdashboard/index.html#/bda7594740fd40299423467b48e9ecf6.

[6] Fehr A.R., Perlman S. (2015). Coronaviruses: An overview of their replication and pathogenesis. Coronaviruses. Methods in Molecular Biology.

[7] Freitas B.T., Durie I.A., Murray J., Longo J.E., Miller H.C., Crich D., Hogan R.J., Tripp R.A., Pegan S.D. (2020). Characterization and Noncovalent Inhibition of the Deubiquitinase and deISGylase Activity of SARS-CoV-2 Papain-Like Protease. ACS Infect. Dis..

[8] Thiel V., Ivanov K.A., Putics A., Hertzig T., Schelle B., Bayer S., Weißbrich B., Snijder E.J., Rabenau H., Doerr H.W. (2003). Mechanisms and enzymes involved in SARS coronavirus genome expression. J. Gen. Virol..

[9] McClain C.B., Vabret N. (2020). SARS-CoV-2: The many pros of targeting PLpro. Signal Transduct. Target. Ther..

[10] Ullrich S., Nitsche C. (2020). The SARS-CoV-2 main protease as drug target. Bioorganic Med. Chem. Lett..

[11] Shin D., Mukherjee R., Grewe D., Bojkova D., Baek K., Bhattacharya A., Schulz L., Widera M., Mehdipour A.R., Tascher G. (2020). Papain-like protease regulates SARS-CoV-2 viral spread and innate immunity. Nature.

[12] Fischer A., Sellner M., Neranjan S., Smieško M., Lill M.A. (2020). Potential Inhibitors for Novel Coronavirus Protease Identified by Virtual Screening of 606 Million Compounds. Int. J. Mol. Sci..

[13] Kazmierski W.M., Hamatake R., Duan M., Wright L.L., Smith G.K., Jarvest R.L., Ji J.J., Cooper J.P., Tallant M.D., Crosby R.M. (2012). Discovery of novel urea-based hepatitis C protease inhibitors with high potency against protease-inhibitor-resistant mutants. J. Med. Chem..

[14] Dzimianski J.V., Scholte F.E., Bergeron É., Pegan S.D. (2019). ISG15: It’s Complicated. J. Mol. Biol..

[15] Barretto N., Jukneliene D., Ratia K., Chen Z., Mesecar A.D., Baker S.C. (2005). The papain-like protease of severe acute respiratory syndrome coronavirus has deubiquitinating activity. J. Virol..

[16] Sulea T., Lindner H.A., Purisima E.O., Ménard R. (2005). Deubiquitination, a new function of the severe acute respiratory syndrome coronavirus papain-like protease?. J. Virol..

[17] Haq S., Ramakrishna S. (2017). Deubiquitylation of deubiquitylases. Open Biol..

[18] Genç B., Jara J.H., Schultz M.C., Manuel M., Stanford M.J., Gautam M., Klessner J.L., Sekerkova G., Heller D.B., Cox G.A. (2016). Absence of UCHL 1 function leads to selective motor neuropathy. Ann. Clin. Transl. Neurol..

[19] Gong B., Cao Z., Zheng P., Vitolo O.V., Liu S., Staniszewski A., Moolman D., Zhang H., Shelanski M., Arancio O. (2006). Ubiquitin hydrolase Uch-L1 rescues *β*-amyloid-induced decreases in synaptic function and contextual memory. Cell.

[20] Darling S., Fielding A.B., Sabat-Pośpiech D., Prior I.A., Coulson J.M. (2017). Regulation of the cell cycle and centrosome biology by deubiquitylases. Biochem. Soc. Trans..

[21] Liu Y., Lashuel H.A., Choi S., Xing X., Case A., Ni J., Yeh L.A., Cuny G.D., Stein R.L., Lansbury P.T. (2003). Discovery of inhibitors that elucidate the role of UCH-L1 activity in the H1299 lung cancer cell line. Chem. Biol..

[22] Virnau P., Mirny L.A., Kardar M. (2006). Intricate knots in proteins: Function and evolution. PLoS Comput. Biol..

[23] Sulkowska J.I. (2020). On folding of entangled proteins: Knots, lassos, links and *θ*-curves. Curr. Opin. Struct. Biol..

[24] Osipiuk J., Azizi S.A., Dvorkin S., Endres M., Jedrzejczak R., Jones K.A., Kang S., Kathayat R.S., Kim Y., Lisnyak V.G. (2021). Structure of papain-like protease from SARS-CoV-2 and its complexes with non-covalent inhibitors. Nat. Commun..

[25] Gao X., Qin B., Chen P., Zhu K., Hou P., Wojdyla J.A., Wang M., Cui S. (2021). Crystal structure of SARS-CoV-2 papain-like protease. Acta Pharm. Sin. B.

[26] Fu Z., Huang B., Tang J., Liu S., Liu M., Ye Y., Liu Z., Xiong Y., Zhu W., Cao D. (2021). The complex structure of GRL0617 and SARS-CoV-2 PLpro reveals a hot spot for antiviral drug discovery. Nat. Commun..

[27] Moustaqil M., Ollivier E., Chiu H.P., Van Tol S., Rudolffi-Soto P., Stevens C., Bhumkar A., Hunter D.J., Freiberg A.N., Jacques D. (2020). SARS-CoV-2 proteases PLpro and 3CLpro cleave IRF3 and critical modulators of inflammatory pathways (NLRP12 and TAB1): Implications for disease presentation across species. Emerg. Microbes Infect..

[28] Kandeel M., Kitade Y., Fayez M., Venugopala K.N., Ibrahim A. (2021). The emerging SARS-CoV-2 papain-like protease: Its relationship with recent coronavirus epidemics. J. Med Virol..

[29] Welker A., Kersten C., Müller C., Madhugiri R., Zimmer C., Müller P., Zimmermann R., Hammerschmidt S., Maus H., Ziebuhr J. (2020). Structure-Activity Relationships of Benzamides and Isoindolines Designed as SARS-CoV Protease Inhibitors Effective against SARS-CoV-2. ChemMedChem.

[30] Rut W., Lv Z., Zmudzinski M., Patchett S., Nayak D., Snipas S.J., El Oualid F., Huang T.T., Bekes M., Drag M. (2020). Activity profiling and crystal structures of inhibitor-bound SARS-CoV-2 papain-like protease: A framework for anti–COVID-19 drug design. Sci. Adv..

[31] Zmudzinski M., Rut W., Olech K., Granda J., Giurg M., Burda-Grabowska M., Zhang L., Sun X., Lv Z., Nayak D. (2020). Ebselen derivatives are very potent dual inhibitors of SARS-CoV-2 proteases-PLpro and Mpro in in vitro studies. bioRxiv.

[32] Leung D., Abbenante G., Fairlie D.P. (2000). Protease inhibitors: Current status and future prospects. J. Med. Chem..

[33] Ratia K., Pegan S., Takayama J., Sleeman K., Coughlin M., Baliji S., Chaudhuri R., Fu W., Prabhakar B.S., Johnson M.E. (2008). A noncovalent class of papain-like protease/deubiquitinase inhibitors blocks SARS virus replication. Proc. Natl. Acad. Sci. USA.

[34] Johnson D.S., Weerapana E., Cravatt B.F. (2010). Strategies for discovering and derisking covalent, irreversible enzyme inhibitors. Future Med. Chem..

[35] Klemm T., Ebert G., Calleja D.J., Allison C.C., Richardson L.W., Bernardini J.P., Lu B.G., Kuchel N.W., Grohmann C., Shibata Y. (2020). Mechanism and inhibition of the papain-like protease, PLpro, of SARS-CoV-2. EMBO J..

[36] Shen Z., Ratia K., Cooper L., Kong D., Lee H., Kwon Y., Li Y., Alqarni S., Huang F., Dubrovskyi O. (2021). Potent, Novel SARS-CoV-2 PLpro Inhibitors Block Viral Replication in Monkey and Human Cell Cultures. bioRxiv.

[37] Weglarz-Tomczak E., Tomczak J.M., Talma M., Brul S. (2020). Ebselen as a highly active inhibitor of PLProCoV2. bioRxiv.

[38] Sargsyan K., Lin C.C., Chen T., Grauffel C., Chen Y.P., Yang W.Z., Yuan H.S., Lim C. (2020). Multi-targeting of functional cysteines in multiple conserved SARS-CoV-2 domains by clinically safe Zn-ejectors. Chem. Sci..

[39] Davies C.W., Chaney J., Korbel G., Ringe D., Petsko G.A., Ploegh H., Das C. (2012). The crystal structure of ubiquitin carboxy-terminal hydrolase L1 (UCHL1) with a tripeptide fluoromethyl ketone (Z-VAE (OMe)-FMK). Bioorganic Med. Chem. Lett..

[40] Boudreaux D.A., Maiti T.K., Davies C.W., Das C. (2010). Ubiquitin vinyl methyl ester binding orients the misaligned active site of the ubiquitin hydrolase UCHL1 into productive conformation. Proc. Natl. Acad. Sci. USA.

[41] Das C., Hoang Q.Q., Kreinbring C.A., Luchansky S.J., Meray R.K., Ray S.S., Lansbury P.T., Ringe D., Petsko G.A. (2006). Structural basis for conformational plasticity of the Parkinson’s disease-associated ubiquitin hydrolase UCH-L1. Proc. Natl. Acad. Sci. USA.

[42] Liu Y., Fallon L., Lashuel H.A., Liu Z., Lansbury P.T. (2002). The UCH-L1 gene encodes two opposing enzymatic activities that affect *α*-synuclein degradation and Parkinson’s disease susceptibility. Cell.

[43] Sułkowska J.I., Rawdon E.J., Millett K.C., Onuchic J.N., Stasiak A. (2012). Conservation of complex knotting and slipknotting patterns in proteins. Proc. Natl. Acad. Sci. USA.

[44] Jamroz M., Niemyska W., Rawdon E.J., Stasiak A., Millett K.C., Sułkowski P., Sulkowska J.I. (2015). KnotProt: A database of proteins with knots and slipknots. Nucleic Acids Res..

[45] Larsen C.N., Price J.S., Wilkinson K.D. (1996). Substrate binding and catalysis by ubiquitin C-terminal hydrolases: Identification of two active site residues. Biochemistry.

[46] Johnston S.C., Larsen C.N., Cook W.J., Wilkinson K.D., Hill C.P. (1997). Crystal structure of a deubiquitinating enzyme (human UCH-L3) at 1.8 å resolution. EMBO J..

[47] Rut W., Zmudzinski M., Snipas S.J., Bekes M., Huang T.T., Drag M. (2020). Engineered unnatural ubiquitin for optimal detection of deubiquitinating enzymes. Chem. Sci..

[48] Wolber G., Langer T. (2005). LigandScout: 3-D pharmacophores derived from protein-bound ligands and their use as virtual screening filters. J. Chem. Inf. Model..

[49] Báez-Santosr Y.M., Barraza S.J., Wilson M.W., Agius M.P., Mielech A.M., Davis N.M., Baker S.C., Larsen S.D., Mesecar A.D. (2014). X-ray structural and biological evaluation of a series of potent and highly selective inhibitors of human coronavirus papain-like proteases. J. Med. Chem..

[50] Wu G., Robertson D.H., Brooks C.L., Vieth M. (2003). Detailed analysis of grid-based molecular docking: A case study of CDOCKER—A CHARMm-based MD docking algorithm. J. Comput. Chem..

[51] Friesner R.A., Banks J.L., Murphy R.B., Halgren T.A., Klicic J.J., Mainz D.T., Repasky M.P., Knoll E.H., Shelley M., Perry J.K. (2004). Glide: A new approach for rapid, accurate docking and scoring. 1. Method and assessment of docking accuracy. J. Med. Chem..

[52] Raies A.B., Bajic V.B. (2016). In silico toxicology: Computational methods for the prediction of chemical toxicity. Wiley Interdiscip. Rev. Comput. Mol. Sci..

[53] Lipinski C.A., Lombardo F., Dominy B.W., Feeney P.J. (1997). Experimental and computational approaches to estimate solubility and permeability in drug discovery and development settings. Adv. Drug Deliv. Rev..

[54] Veber D.F., Johnson S.R., Cheng H.Y., Smith B.R., Ward K.W., Kopple K.D. (2002). Molecular properties that influence the oral bioavailability of drug candidates. J. Med. Chem..

[55] (2020). LigandScout 4.4.5.

[56] (2020). BIOVIA Discovery Studio v20.1.0.19295.

[57] (2016). Statistica 13.1.

[58] (2017). Schrödinger Release 2017-1.

[59] Chen J.H., Linstead E., Swamidass S.J., Wang D., Baldi P. (2007). ChemDB update—Full-text search and virtual chemical space. Bioinformatics.

