# Supplementary material for: SARS-CoV-2 Papain-Like Protease Potential Inhibitors—In Silico Quantitative Assessment
Source: Int J Mol Sci. 2021 Apr 12;22(8):3957. doi: 10.3390/ijms22083957 (PMC8069282; doi:10.3390/ijms22083957)
Supplement: Supplementary file 1 [file ijms-22-03957-s001.pdf]

Supplementary Materials: SARS-CoV-2 Papain-Like Protease Potential Inhibitors—In Silico Quantitative Assessment

Adam Stasiulewicz [1,2], Alicja W. Maksymiuk [1,3], Mai Lan Nguyen [1,4], Barbara Bełza [1,5] and Joanna I. Sulkowska [1,6,*]

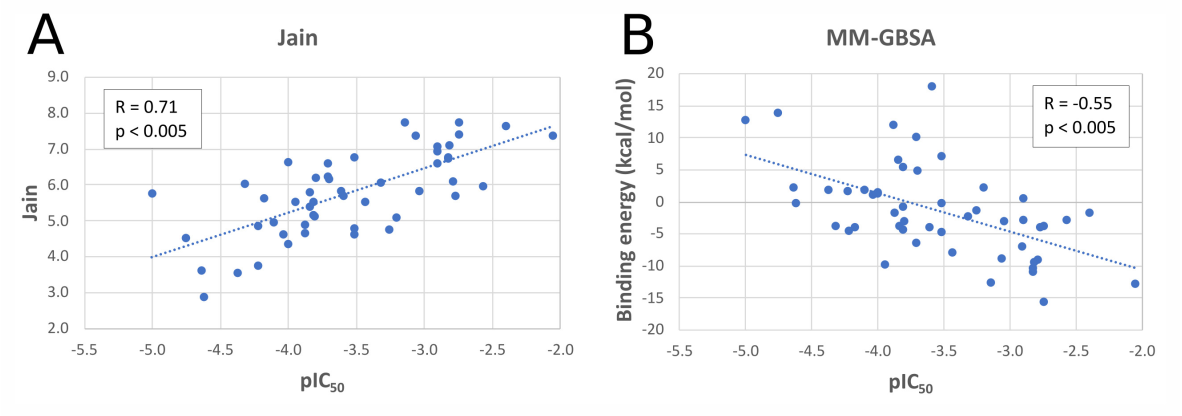

Figure S1. Correlation between values of scoring functions and binding energies, and pIC_{50} values of the extended set of inhibitors docked to PLpro (PDB ID: 7jn2). (**A**) Jain scoring function. (**B**) MM–GBSA.

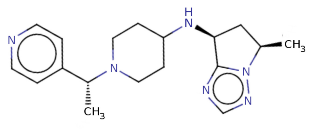
1

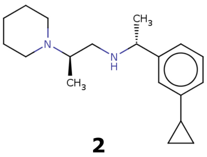
2

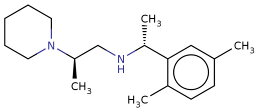
3

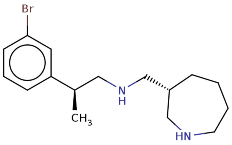
4

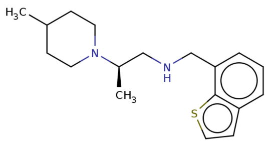
5

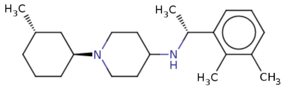
6

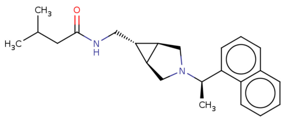
7

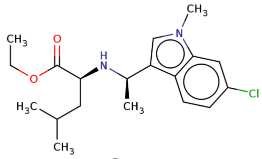
8

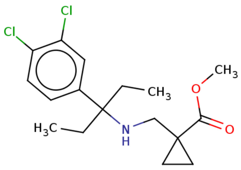
9

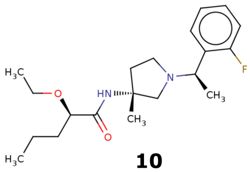
10

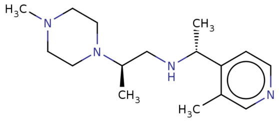
11

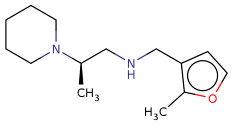
12

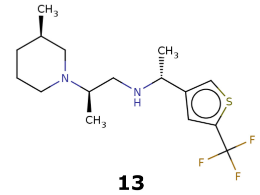
13

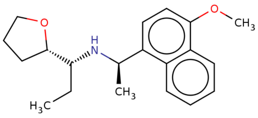
14

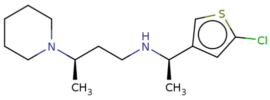
15

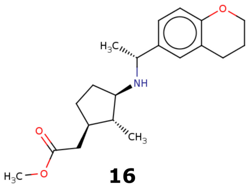
16

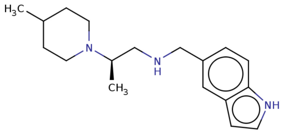
17

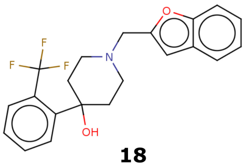
18

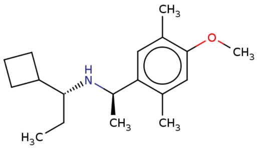
19

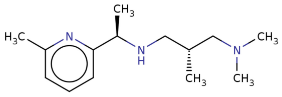
20

Figure S2. Structural formulas of the 20 potentially selective PLpro inhibitors candidates with the lowest predicted IC_{50} values based on the MLR model estimation.

Table 1. Summary of SARS-CoV-2 PLpro crystal structures available in the PDB database.

PDB ID	Ligand name	Ligand type	Resolution [Å]	Mutations	IC_{50} [μM]	References
6wrh	-	-	1.60	C111S	-	[1]
6wzu	-	-	1.79	-	-	[1]
6xg3	-	-	2.48	C111S	-	[1]
7cjd	-	-	2.50	C111S	-	[2]
6w9c	-	-	2.70	-	-	-
7d47	-	-	1.97	C111S		-
7d6h	-	-	1.60	C111S	-	-
7nfv	-	-	1.42	-	-	-
6xaa	ubiquitin	-	2.70	C111S	-	[3]
6yva	ISG15	-	3.18	C111S	-	[4]
6xa9	ISG15	-	2.90	-	-	[3]
6wuu	VIR250	covalent	2.79	-	-	[5]
6wx4	VIR251	covalent	1.66	-	-	[5]
7jir	GRL0617	non-covalent	2.09	C111S	2.30	[1]
7jit	PLP_Snyder495	non-covalent	1.95	C111S	5.10	[1]
7jiv	PLP_Snyder530	non-covalent	2.05	C111S	6.40	[1]
7jiw	PLP_Snyder530	non-covalent	2.30	-	6.40	[1]
7cmd	GRL0617	non-covalent	2.59	-	2.20	[2]
7cjm	GRL0617	non-covalent	3.20	C111S	2.20	-
7jrn	GRL0617	non-covalent	2.48	-	2.20	-
7jn2	PLP_Snyder441	non-covalent	1.93	-	-	-
7koj	PLP_Snyder494	non-covalent	2.02	C111S	-	-
7kok	PLP_Snyder496	non-covalent	2.00	C111S	-	-
7kol	PLP_Snyder496	non-covalent	2.58	-	-	-
7krx	PLP_Snyder441	non-covalent	2.72	C111S	-	-
7lbr	XR8-89	non-covalent	2.20	-	0.11	[6]
7lbs	XR8-24	non-covalent	2.80	-	0.56	[6]
7llf	XR8-83	non-covalent	2.30	-	0.21	[6]
7llz	XR8-69	non-covalent	2.90	-	0.37	[6]
7los	XR8-65	non-covalent	2.90	-	0.33	[6]
7e35	S43	non-covalent	2.40	C111S	-	-
7m1y	ebselen	-	2.02	C111S	-	-

Table 2. Cross-docking ligand heavy-atom RMSD [Å] after superposition of proteins by Cα atoms. Redocking results are presented in green. If the PDB structure had more than one protein chain, the chain chosen in this table has its name added after underscore. 7jir, 7jrn, 7cjm, and 7cmd have the same inhibitor so only the results of 7jir ligand docking are presented. The same applies to 7jiw and 7jiv. Two endmost ligands come from SARS-CoV PLpro.

	Protein structure										
Ligand from	7jir	7jrn_A	7cjm	7cmd_D	7jit	7jiv	7jiw	6wuu_A	6wx4	7jn2	6w9c_A
7jir	0.4	2.7	0.8	0.6	0.4	0.6	0.3	6.7	7.5	0.5	1.3
7jit	1.8	1.7	1.0	1.1	1.2	1.0	1.0	6.2	6.5	2.8	4.5
7jiv	0.5	0.8	1.2	0.6	0.5	0.3	0.3	4.7	4.9	4.2	8.1
6wuu	8.0	8.3	9.3	8.7	8.7	8.0	8.6	1.1	2.5	6.0	9.7
6wx4	9.9	9.8	7.8	9.9	8.4	8.3	8.0	2.6	2.0	6.0	9.7
7jn2	1.6	1.8	1.7	1.7	0.5	2.9	2.8	4.0	6.8	1.6	1.8
4ovz	2.9	3.4	3.5	3.5	2.8	3.0	2.8	8.0	8.8	3.1	2.8
4ow0	3.0	3.6	9.2	3.7	2.9	2.9	2.9	7.9	8.3	3.3	6.6

Table 3. Pearson correlation coefficients between pIC_{50} values of inhibitors and values of scoring functions and MM–GBSA listed in the first column. Abbreviations: E.—Energy, I.E.—Interaction Energy. Top nine functions are expected to have positive correlations, whereas MM–GBSA is expected to be negatively correlated. Values above 0.5 or below –0.5 are shown in green. If the PDB structure has more than one chain, the chain chosen in this table has its name added after underscore. 7jit and 7jiv have a C111S mutation, and 7jit_Cys and 7jiv_Cys have the serine residue mutated back into cysteine.

	7jiw	6w9c_A	7jn2	7jit	7jit_Cys	7jiv	7jiv_Cys	7cmd_D	7jrn_A
–CDOCKER E.	0.13	0.17	0.16	0.08	0.17	0.17	0.12	0.11	0.18
–CDOCKER I.E.	0.46	0.60	0.56	0.38	0.45	0.45	0.46	0.55	0.62
LigScore1	0.19	0.38	0.22	0.10	0.18	0.18	0.16	0.17	0.24
LigScore2	0.19	0.31	0.22	0.10	0.17	0.17	0.11	0.10	0.26
–PLP1	0.06	0.26	0.25	0.02	0.16	0.16	0.06	0.21	0.39
–PLP2	0.14	0.32	0.31	0.00	0.25	0.25	0.16	0.27	0.41
Jain	0.53	0.53	0.73	0.39	0.43	0.43	0.44	0.65	0.23
–PMF	0.39	0.12	0.37	0.49	0.45	0.45	0.46	0.21	0.36
–PMF04	0.39	0.21	0.44	0.51	0.47	0.47	0.42	0.14	0.29
MM–GBSA	-0.66	−0.54	−0.64	−0.50	−0.56	−0.64	−0.64	−0.53	−0.66

Table 4. Cross-docking results for 7jn2. Ligands from the PDB entries in the first column were docked to the 7jn2 PLpro structure. Proteins were superimposed by Cα atoms and ligand heavy-atom RMSD [Å] was calculated. Redocking results are presented in green. 6wuu and 6wx4 have covalent inhibitors. Two endmost ligands come from SARS-CoV PLpro.

Ligand from	RMSD [Å]
7jn2	1.6
7jir	0.5
7jit	2.8
7jiv	4.2
7koj	1.5
7kok	1.2
7lbr	1.1
7lbs	1.3
7llf	4.5
7llz	4.3
7los	1.7
7e35	3.6
6wuu	6.0
6wx4	6.0
4ovz	3.1
4ow0	3.3

Table 5. Performance of the selected pharmacophore for different numbers of omitted features. The value of 14 was selected for the drug screening.

Max. Number of Omitted Features	Hits	True positives	$EF_{1\%}$
17	574	6	67.0
16	569	6	67.0
15	288	5	67.0
14	110	5	67.0
13	24	5	67.0
12	6	4	111.7

Table 6. SARS-CoV-2 PLpro inhibitors used for the confirmatory validation of the best pharmacophore. Names in the first column reflect the names used in the cited sources.

Compound	IC_{50} [nM]	References
Jun9-72-2	670	[7]
Jun9-75-4	620	
Jun9-75-5	560	
Jun9-84-3	670	
DY-3-15	800	[6]
XR8-23	390	
XR8-24	560	
XR8-30	750	
XR8-32-2	810	
XR8-57	700	
XR8-65	330	
XR8-67	170	
XR8-79	410	
XR8-83	210	
XR8-89	113	
XR8-98	810	
ZN-2-185	600	
ZN-2-187	800	
ZN-3-80	590	
rac5c_R	810	[3]

Table 7. PLpro inhibitors used for validation of binding affinity prediction. Names in the first column reflect the names used in the cited sources.

Compound	IC_{50} [nM]	Description	References
2	5100	GRL0617 derivatives	[1]
3	6400		
4	43200		
5	16800		
6	7000		
7	12700		
2a	6900	GRL0617 derivatives	[8]
2i	15000		
2n	42000		
2p	7500		
2r	10000		
2s	10000		
14h	7600		
14k	57000		
6577871	100700	similar to the ligand from 4ovz	[9]
7724772_R	23500	GRL0617 without the amine group	
7724772_S		S enantiomer	
Compound 6	5000	GRL0617 amide, R enantiomer	
GRL0617	2100	ligand from 7jir	[10]
rac3j_R	1400	ligand from 4ovz	[3]
rac3j_S		S enantiomer	
rac3k_R	1150	ligand from 4ow0	
rac3k_S		S enantiomer	
rac5c_R	810	similar to the ligand from 4ow0 and 4ovz	
rac5c_S		S enantiomer	

Table 8. PLpro inhibitors used for additional validation of binding affinity prediction. Names in the first column reflect the names used in the cited sources.

Compound	IC_{50} [nM]	References
Jun9-67-1	2720	[7]
Jun9-68-3	5130	
Jun9-72-2	670	
Jun9-75-3	8890	
Jun9-75-4	620	
Jun9-75-5	560	
Jun9-84-3	670	
Jun9-85-1	660	
Jun9-85-5	16590	
Jun9-86-2	6490	
DY-3-15	800	[6]
DY-3-65	6300	
DY-3-66	3300	
DY2-109	21000	
DY2-137	3300	
XDY2-62	3300	
XR8-24	560	
XR8-61	6500	
XR8-69	370	
XR8-89	113	
XR8-9	1800	
XR8-96	250	
ZN-2-181	1100	
ZN-2-187	800	
ZN-2-188-1	1600	
ZN-3-56	3900	
ZN-3-66	4100	
ZN-3-71	10900	
ZN-3-80	590	

Table 9. Summary of compounds with known in vitro activity against UCH-L1 used for molecular docking validation conducted in Maestro 2017-1. ΔG_{bind} values show MM–GBSA binding free energies calculated for ligands docked to the UCH-L1 structure (PDB ID: 4jkj, chain B) using Glide SP.

Compound	IC_{50} [nM]	ΔG_{bind} [kcal/mol]	References
1	6000	−42.78	[11]
3	12000	−39.12	
5	17000	−48.80	
8	51000	−44.01	
9	290000	−19.22	
10	126000	−26.29	
11	75000	−20.77	
13	3400	−40.99	
14	3400	−43.96	
20	36000	−43.39	
30	880	−43.07	
31	1200	−41.71	
33	1300	−43.93	
34	1800	−46.37	
38	9500	−28.85	
41	16000	−35.46	
42	19000	−40.20	
43	19000	−35.70	
45	52000	−38.15	
46	95000	−36.92	
50	810	−46.00	
51	940	−46.45	
53	2900	−45.52	
57	12000	−35.96	
59	12000	−45.55	
60	15000	−41.12	
61	16000	−48.76	
63	50000	−40.56	
B2	15000	−28.13	[12]
Vialinin A	22300	−30.07	[13]

Abbreviations

The following abbreviations are used in this manuscript:

EF	enrichment factor
IC_{50}	half maximal inhibitory concentration
ISG15	interferon-stimulated gene 15
MLR	multiple linear regression
MM–GBSA	molecular mechanics–generalized Born and surface area solvation
PDB	Protein Data Bank
PLpro	papain-like protease
RMSD	root-mean-square deviation
SARS-CoV	severe acute respiratory syndrome coronavirus
SP	standard precision
UCH-L1	ubiquitin carboxy-terminal hydrolase L1

References

1. Osipiuk, J.; Azizi, S.A.; Dvorkin, S.; Endres, M.; Jedrzejczak, R.; Jones, K.A.; Kang, S.; Kathayat, R.S.; Kim, Y.; Lisnyak, V.G.; et al. Structure of papain-like protease from SARS-CoV-2 and its complexes with non-covalent inhibitors. *Nat. Commun.* **2021**, *12*, 1–9.
2. Gao, X.; Qin, B.; Chen, P.; Zhu, K.; Hou, P.; Wojdyla, J.A.; Wang, M.; Cui, S. Crystal structure of SARS-CoV-2 papain-like protease. *Acta Pharm. Sin. B* **2021**, *11*, 237–245.
3. Klemm, T.; Ebert, G.; Calleja, D.J.; Allison, C.C.; Richardson, L.W.; Bernardini, J.P.; Lu, B.G.; Kuchel, N.W.; Grohmann, C.; Shibata, Y.; et al. Mechanism and inhibition of the papain-like protease, PLpro, of SARS-CoV-2. *EMBO J.* **2020**, *39*, e106275.
4. Shin, D.; Mukherjee, R.; Grewe, D.; Bojkova, D.; Baek, K.; Bhattacharya, A.; Schulz, L.; Widera, M.; Mehdipour, A.R.; Tascher, G.; et al. Papain-like protease regulates SARS-CoV-2 viral spread and innate immunity. *Nature* **2020**, *587*, 657–662.
5. Rut, W.; Lv, Z.; Zmudzinski, M.; Patchett, S.; Nayak, D.; Snipas, S.J.; El Oualid, F.; Huang, T.T.; Bekes, M.; Drag, M.; et al. Activity profiling and crystal structures of inhibitor-bound SARS-CoV-2 papain-like protease: A framework for anti–COVID-19 drug design. *Sci. Adv.* **2020**, *6*, eabd4596.
6. Shen, Z.; Ratia, K.; Cooper, L.; Kong, D.; Lee, H.; Kwon, Y.; Li, Y.; Alqarni, S.; Huang, F.; Dubrovskyi, O.; et al. Potent, Novel SARS-CoV-2 PLpro Inhibitors Block Viral Replication in Monkey and Human Cell Cultures. *bioRxiv* **2021**.
7. Xia, Z.; Sacco, M.; Ma, C.; Townsend, J.; Kitamura, N.; Hu, Y.; Ba, M.; Szeto, T.; Zhang, X.; Meng, X.; et al. Discovery of SARS-CoV-2 papain-like protease inhibitors through a combination of high-throughput screening and FlipGFP-based reporter assay. *bioRxiv* **2021**.
8. Welker, A.; Kersten, C.; Müller, C.; Madhugiri, R.; Zimmer, C.; Müller, P.; Zimmermann, R.; Hammerschmidt, S.; Maus, H.; Ziebuhr, J.; et al. Structure-Activity Relationships of Benzamides and Isoindolines Designed as SARS-CoV Protease Inhibitors Effective against SARS-CoV-2. *ChemMedChem* **2020**, *15*, 1–16.
9. Freitas, B.T.; Durie, I.A.; Murray, J.; Longo, J.E.; Miller, H.C.; Crich, D.; Hogan, R.J.; Tripp, R.A.; Pegan, S.D. Characterization and Noncovalent Inhibition of the Deubiquitinase and deISGylase Activity of SARS-CoV-2 Papain-Like Protease. *ACS Infect. Dis.* **2020**, *6*, 2099–2109.
10. Fu, Z.; Huang, B.; Tang, J.; Liu, S.; Liu, M.; Ye, Y.; Liu, Z.; Xiong, Y.; Cao, D.; Li, J.; et al. Structural basis for the inhibition of the papain-like protease of SARS-CoV-2 by small molecules. *bioRxiv* **2020**.
11. Liu, Y.; Lashuel, H.A.; Choi, S.; Xing, X.; Case, A.; Ni, J.; Yeh, L.A.; Cuny, G.D.; Stein, R.L.; Lansbury Jr, P.T. Discovery of inhibitors that elucidate the role of UCH-L1 activity in the H1299 lung cancer cell line. *Chem. Biol.* **2003**, *10*, 837–846.
12. Mitsui, T.; Hirayama, K.; Aoki, S.; Nishikawa, K.; Uchida, K.; Matsumoto, T.; Kabuta, T.; Wada, K. Identification of a novel chemical potentiator and inhibitors of UCH-L1 by in silico drug screening. *Neurochem. Int.* **2010**, *56*, 679–686.
13. Okada, K.; Ye, Y.Q.; Taniguchi, K.; Yoshida, A.; Akiyama, T.; Yoshioka, Y.; Onose, J.I.; Koshino, H.; Takahashi, S.; Yajima, A.; et al. Vialinin A is a ubiquitin-specific peptidase inhibitor. *Bioorg. Med. Chem. Lett.* **2013**, *23*, 4328–4331.
